# DNA Methylation Age Drift Is Associated with Poor Outcomes and De-Differentiation in Papillary and Follicular Thyroid Carcinomas

**DOI:** 10.3390/cancers13194827

**Published:** 2021-09-27

**Authors:** Tiantian Liu, Jiansheng Wang, Yuchen Xiu, Yujiao Wu, Dawei Xu

**Affiliations:** 1 Pathology Department, School of Basic Medical Science, Shandong University, Jinan 250012, China; wangjiansheng222@foxmail.com (J.W.); sirxyc1996@126.com (Y.X.); 2 Department of Medicine, Division of Hematology, Bioclinicum and Center for Molecular Medicine (CMM), Karolinska University Hospital Solna, Karolinsk Institutet, SE-171 76 Stockholm, Sweden; Yujiao.Wu@ki.se (Y.W.); dawei.xu@ki.se (D.X.)

**Keywords:** aging, DNA methylation age, epigenetic clock, prognosis, thyroid carcinoma

## Abstract

**Simple Summary:**

Normal human tissues contain an epigenetic clock resulting from the age-dependent DNA methylation signature, which is the so-called DNA methylation (DNAm) age and can be used to precisely predict chronological age of healthy individuals. Abnormal DNAm age drift has been implicated in cancer risk and pathogenesis. Here, we observed that highly drifted DNAm age (HDDA) occurred in approximately 1/3 tumors derived from patients with papillary and follicular thyroid carcinomas. HDDA is significantly associated with dedifferentiation of tumor cells and poor patient outcomes. Thus, HDDA may serve as a new prognostic factor for thyroid carcinoma.

**Abstract:**

Alterations in global DNA methylation play a critical role in both aging and cancer, and DNA methylation (DNAm) age drift has been implicated in cancer risk and pathogenesis. In the present study, we analyzed the TCGA cohort of papillary and follicular thyroid carcinoma (PTC and FTC) for their DNAm age and association with clinic-pathological features. In 54 noncancerous thyroid (NT) samples, DNAm age was highly correlated with patient chronological age (R^2^ = 0.928, *p* = 2.6 × 10^−31^), but drifted to younger than chronological age in most specimens, especially those from patients >50 years old. DNAm age in 502 tumors was also correlated with patient chronological age, but to a much lesser extent (R^2^ = 0.403). Highly drifted DNAm age (HDDA) was identified in 161 tumors, among which were 101 with DNAm age acceleration while 60 with DNAm age deceleration. Tumors with HDDA were characterized by the robust aberrations in metabolic activities, extracellular microenvironment components and inflammation/immunology responses, and dedifferentiation. Importantly, HDDA in tumors independently predicted shorter disease-free survival of patients. Collectively, NT thyroids from TC patients have younger DNAm age, while HDDA frequently occurs in TCs, and contributes to the TC progression and poor patient outcomes. HDDA may serve as a new prognostic factor for TCs.

## 1. Introduction

Alterations in DNA methylation occur in an age-dependent manner [1], and accordingly, the DNA methylation (DNAm) age or epigenetic clock model has been established to measure chronological age [1,2,3]. One of such seminal models, developed by Horvath, shows that 353 CpGs, differentially methylated during aging, serve as a robust predictor for chronological age in multiple human tissues/organs [2]. Many environmental factors or lifestyles may significantly affect age-related DNA methylation, thereby leading to DNAm age acceleration or deceleration (DNAmaa or DNAmad) [1], which is collectively called DNAm age drift [4]. For instance, DNAmaa is frequently observed in obese or smoking populations [5,6]. Therefore, DNAm age are thought to represent “biological” age. Consistently, DNAmaa has been shown to reliably predict age-related morbidity and mortality [7,8,9,10,11]. Because genome-wide DNA methylation studies show shared epigenomic features between aging and cancer, providing explanations for their possible molecular links [12,13,14], the role for DNAm age in carcinogenesis has been recently explored. The obtained results indeed indicate that DNAmaa increases cancer risk, promote cancer initiation or progression, and predict poor patient outcomes in different types of cancer [10,15,16,17,18,19,20,21,22]. However, in some other cancers, DNAmad is associated with aggressive diseases and shorter survival. Therefore, DNAm age drift, either DNAmaa or DNAmad, may be involved in carcinogenesis in context-dependent manners.

Thyroid carcinoma (TC) is the commonest endocrine malignancy, and its incidence has significantly increased in the past three decades [23]. Tumors transformed from follicular thyroid cells account for >95% of all TCs, while the vast majority of them belong to differentiated papillary TCs (PTCs, up to 85%) and follicular TCs (FTCs, 10–15%) [24]. Recent genomic and epigenetic analyses have gained profound insights into the pathogenesis of these TCs [25,26,27], but there is still great need for improvements in risk stratification, prognostication, treatment decision and identification of new therapeutic targets [24]. Like other human malignancies, TCs exhibit widespread aberrations of DNA methylation [25,26,27]. However, little has been known about the relationship between DNAm age and clinical characteristics in TCs. In the present study, we thus determined whether the DNAm age drift was involved in the TC formation/progression and has a clinical implication in the disease prognosis by analyzing the TCGA cohort of TC patients.

## 2. Materials and Methods

### 2.1. Study Subjects/Specimens, DNA Methylation and Expression Data

The study includes 502 patients with TC in the TCGA database [25]. For 54 patients, both tumors and matched non-tumorous thyroid (NT) specimens were analyzed. Clinical and pathological information, and DNA methylation (Illumina 450 K platform) data were downloaded from The Cancer Genome Atlas (TCGA) Legacy Archieve (http://cancergenome.nih.gov, accessed on 7 June 2021). DNA methylation data are available in a total of 502 TC tumors, and 54 NT specimens. In addition, RNA sequencing data from those tumors were also downloaded.

### 2.2. Calculation for DNAm Age, DNAm Age Acceleration (DNAmaa) and Deceleration (DNAmad)

The Horvath model was introduced in 2013 to predict chronological age by determining the methylation of 353 CpGs in various types of tissues. Briefly, these 353 CpGs are selected using a penalized regression model, and 190 of them get hypermethylated while 160 hypomethylated with increased age. DNAm age was calculated according to the methylation beta values of 353 CpGs [2], which is available at https://dnamage.genetics.ucla.edu, accessed on 15 June 2021. The following formula was used for calculation:

DNAmAge = inverse: F(α_0_ + α_1_CpG_1_ + …+ α_353_CpG_353_).

where F is a function for transformation of age and α_i_s are coefficients generated from the elastic net regression model. The calculation accuracy was evaluated using the median absolute difference (MAD) between DNAm and chronological age. DNAmaa or DNAmad is simply expressed as a deviation between chronological age and DNAm age, or residual of DNAm age extracted from chronological age.

In addition, for differences in global DNA methylation among groups, differential methylation probes (DMPs) were sorted out using |Δβ|> 0.06 and adjusted *p* < 0.05.

### 2.3. Differentially Expressed Genes (DEGs) and Gene Ontology (GO) Term/Pathway Enrichment Analyses

DEGs between tumors with and without HDDA were identified using edgeR packages in R software. An adjusted *p* < 0.05 and fold change (log2) > 1.5 were considered as statistically significant. GO term enrichment analysis maps a set of genes/gene products from three aspects: cellular components, biological process and molecular function. The Kyoto Encyclopedia of Genes and Genomes (KEGG) pathway enrichment analyses were performed to explore alterations in signaling pathways in tumors with HDDA.

### 2.4. Thyroid Differentiation Score (TDS)

TDS in each tumor sample was calculated based on mRNA expression levels within selected set of 12 thyroid function genes (TG, TPO, SLC26A4, SLC5A5, SLC5A8, DIO1, DIO2, DUOX1, DUOX2, KIT, TFF3 and FHL1) using the following formula: TDS = Mean of Log2(Fold Change) across 12 genes. The association between TDS and DNAm age drift was then evaluated.

### 2.5. Statistical Analyses

Based on the distribution of data, Student’s *t*-test, Mann–Whitney *U*-test, and Chi^2^-test or Fisher’s exact test were used for analysis. Pearson’s correlation coefficient was applied to calculate correlation coefficients R^2^. Survival analyses were performed with log-rank test. Overall and disease-free survivals (OS and DFS) were visualized with Kaplan-Meier plots. Multivariate analysis was performed with a Cox regression model.

## 3. Results

### 3.1. Clinic-Pathological Characteristics of the TCGA Cohort of Patients with TC

The clinic-pathological data for 508 TC patients were downloaded from TCGA Legacy Archieve (http://cancergenome.nih.gov/, accessed on 7 June 2021) and patient characteristics were summarized in Table 1. In this cohort, PTC and FTC numbers were 399 and 107, respectively, covering 99.6% of the total patients.

### 3.2. Correlation between Chronological Age and DNAm Age in NT Specimens and TC Tumors

**Figure 1:**
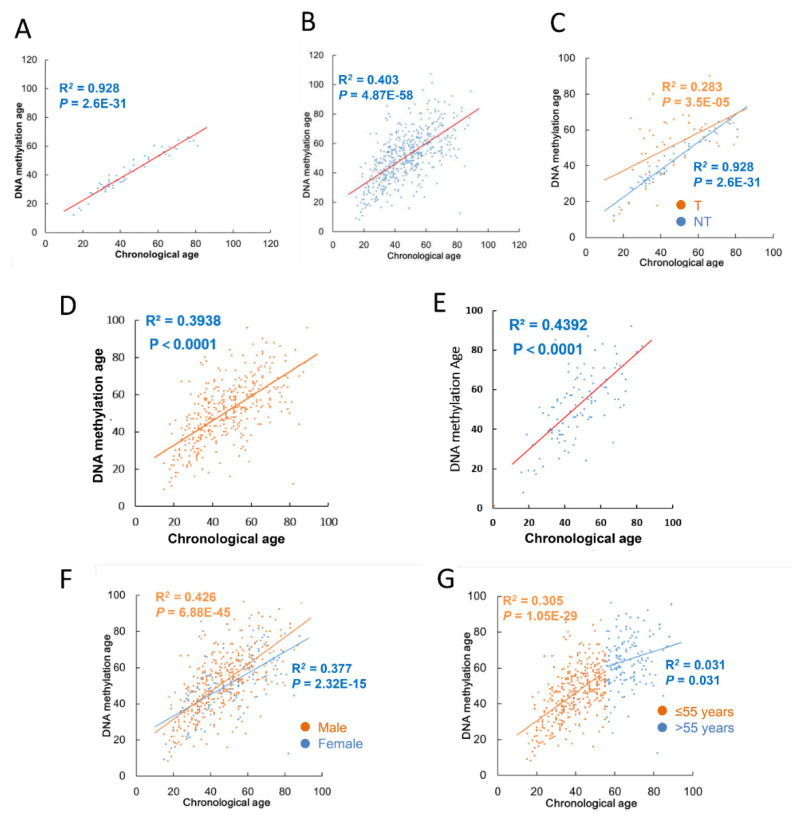
The correlation between DNAm and chronological age in noncancerous thyroid (NT) tissues and tumors from patients with thyroid carcinoma (TC) in the TCGA cohort. (**A**) The high correlation between DNAm and chronological age in NTs from 54 patients with TC. (**B**) The correlation between DNAm and patient chronological age in 502 TC tumors. (**C**) The correlation between DNAm and chronological age in 54 NTs and their matched TC tumors (T) from the same patients. (**D**) The correlation between DNAm and chronological age in PTC tumors. (**E**) The correlation between DNAm and chronological age in FTC tumors. (**F**) The correlation between DNAm and chronological age in TC tumors from male and female patients. (**G**) Weak correlation between DNAm and chronological age in TC tumors from patients with >55 years old.

In 508 TC patients above, tumors from 502 of them were analyzed for global DNA methylation using Illumina 450 K platform. In addition, DNA methylation profiling was also performed on NT specimens from 54 patients. We thus analyzed DNAm age in 54 NTs and 502 TC tumors and compared it with patient chronological age. For NTs, chronological age is highly correlated with DNAm age (R^2^ = 0.928 with MAD = −3 years; range (median): 12–66 (38)) (Figure 1A and Table 2), consistent with the earlier report [2]. Unexpectedly, 37 of 54 NTs displayed younger DNAm age than their chronological age, or DNAm age deceleration, which was even more evident in patients >50 years old (21/22) (Table S1). The analyses of 502 TC tumors also showed a significant correlation between DNAm and chronological age (R^2^ = 0.41; range (median): 8–121 (51)) (Figure 1B and Table 2), but to a much lesser extent compared to that in NTs. Figure 1C further illustrates such a difference by placing NTs and all their matched tumors from the same patients together. In addition, in patients whose DNAm age in NTs was younger or older than their chronological age, their matched tumors usually exhibited similar DNAm age drift (Table S1). The separate analysis of PTC and FTC tumors showed largely same correlations between chronological and DNAm age (Figure 1D,E, and Table 2). There is no significant difference in DNAm age drift between male and female patients (Figure 1F).

### 3.3. DNAmaa and DNAmmad in TC Tumors

DNAm age drift including DNAmaa and DNAmad was further analyzed in TC tumors. In those 502 tumors, DNAm age was exactly matched with their chronological age in 17 patients, while 304 and 181 of them exhibited DNAmaa (+1 to +55, median absolute difference (MAD) = 10) and DNAmad (−1 to −70, MAD = −8 years), respectively. Of note, for tumors derived from TC patients with chronological age >55, the correlation with DNAm age was almost not observed any longer (Figure 1G). The vast majority of TC tumors derived from the patient group >55 years had either DNAmaa or DNAmad. These findings suggest widespread disruptions of the DNAm age signature in TC tumors from older patients.

### 3.4. DNAm Age Drift as a Prognostic Factor for TC Patients

**Figure 2:**
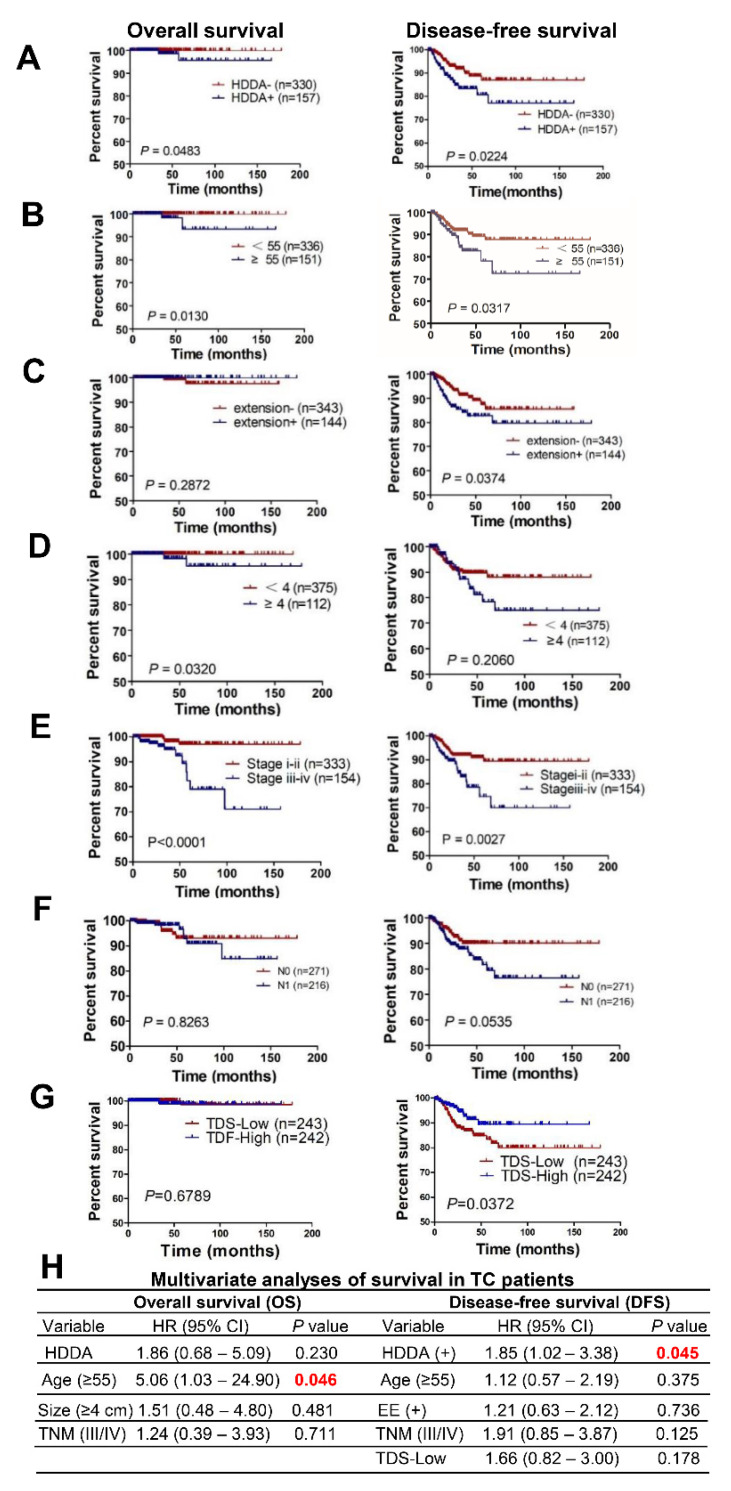
Highly drifted DNAm age (HDDA) independently predicts shorter patient disease-free survival (DFS) in TCs. (**A**–**G**) HDDA, chronological age, extrathyroidal extension, tumor size, N and TNM stages, and thyroid differentiation score (TDS) for their association with OS and DFS, respectively. (**H**) Multivariate analyses of the variables above for association with OS and DFS. EE: Extrathyroidal extension. P values in red: Statistically significant.

DNAmaa or DNAmad has been shown as a prognostic factor in several types of cancer, and we thus sought to determine whether this is the case in TCs, too. For this purpose, three terciles were used to categorize 502 patients into low, middle and high groups for DNAmaa and DNAmad, respectively, based on their MADs. The total number of tumors with high DNAmaa and high DNAmad were 101 and 60, respectively, which was collectively named as the group highly drifted DNAm age (HDDA). A total of 487 patients in the TCGA cohort have OS and DFS data. For both OS and DFS, the HDAA group had significantly shorter survival than did other patients (Figure 2A) (*p* = 0.0483 and 0.0224 for OS and DFS, respectively). Because age (>55 years), tumor size (>4 cm), extrathyroidal extension, N and TNM stages, and thyroid differentiation score (TDS) are known TC prognostic factors, we further analyzed the influence of these factors on patient survival. Indeed, all these variables except N stage predicted patient OS and/or DFS in this cohort of TCs (Figure 2B–G). However, multivariate analyses showed that only age >55 was significantly associated with shorter patient OS (*p* = 0.046), whereas the presence of HDDA was the only one independently predicting DFS with a statistical significance (*p* = 0.045) (Figure 2H). There was a tendency for TNM stages III/IV to be associated with shorter DFS with *p* value 0.125. In addition, there was no difference in HDDA between tumors at stage I/II and III/IV (*p* = 0.678), further supporting its independent prognostic value for DFS in this cohort of patients.

We further evaluated the effect of HDDA on survival of PTC and FTC patients separately. The presence of HDDA was significantly associated with OS in PTC patients (*p* = 0.045), while its association with DFS did not reach a statistical significance (at the border line, *p* = 0.060) (Figure S1A). However, for a 10-year DFS, 241 of 263 patients without HDDA whereas only 101 of 119 HDDA patients remained disease-free (Chi^2^-test: *p* = 0.044), suggesting that HDDA influences long-term survival more significantly in PTC. For FTC tumors with HDDA, there was only a tendency to have shorter OS and DFS (Figure S1B), similar for the 10-year DFS (*p* = 0.226).

### 3.5. Alterations in Global DNA Methylation in TC Tumors with HDDA

**Figure 3:**
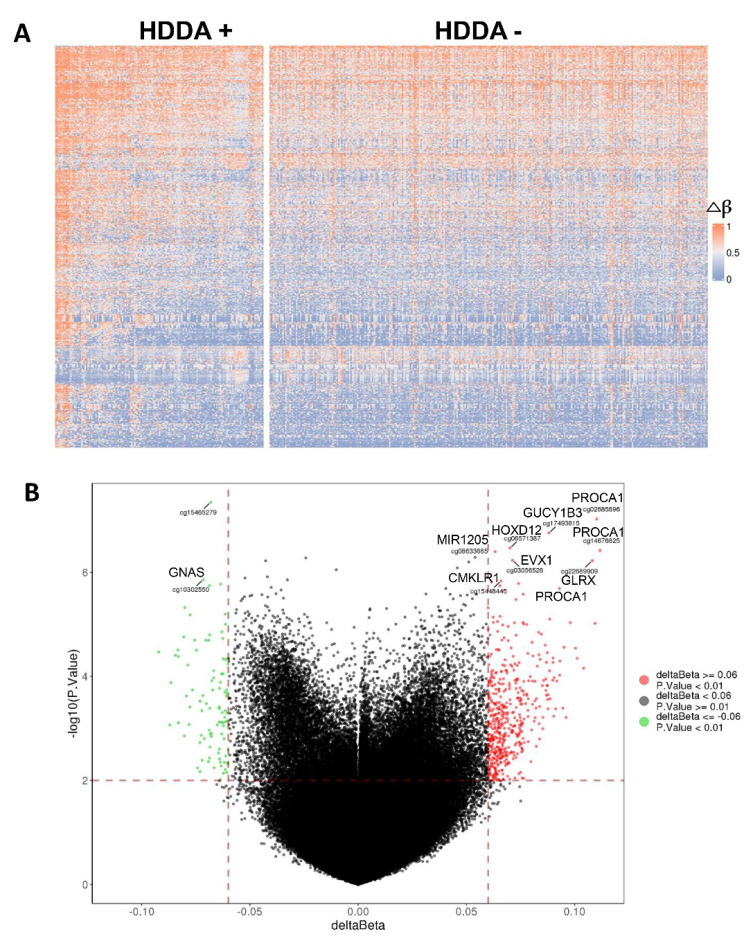
Differentially methylated probes (DMPs) between TC tumors with and without HDDA. (**A**) The heatmap of DMPs in tumors with and without HDDA based on 309 DMPs in the Table S2. DMPs with |Δβ| > 0.06 and adjusted *p* < 0.05 were included. The scale is from 0 (blue) to 1 (red). (**B**) The volcano image showing the most significant DMPs and genes where they reside. Green dots: significantly hypomethylated probes (57) in HDDA tumors, and red dots: significantly hypermethylated probes (252) in HDDA tumors (compared to tumors without HDDA).

The difference in global DNA methylation was further compared between patients with and without HDDA, and a total of 309 DMPs, which were unrelated with DNAm age, were identified (Table S2, Figure 3A). Compared to the TC tumors without HDDA, 57 of 309 DMPs were hypomethylated while 252 hypermethylated. PROCA1, GUCY1B3, EVX1, GLRX, MR1205, CMKLR1 and GNAS were observed as the genes harboring DMPs with the most significance (Figure 3B).

### 3.6. DEGs and GO Term/Pathway Enrichments in TC Tumors with HDDA

**Figure 4:**
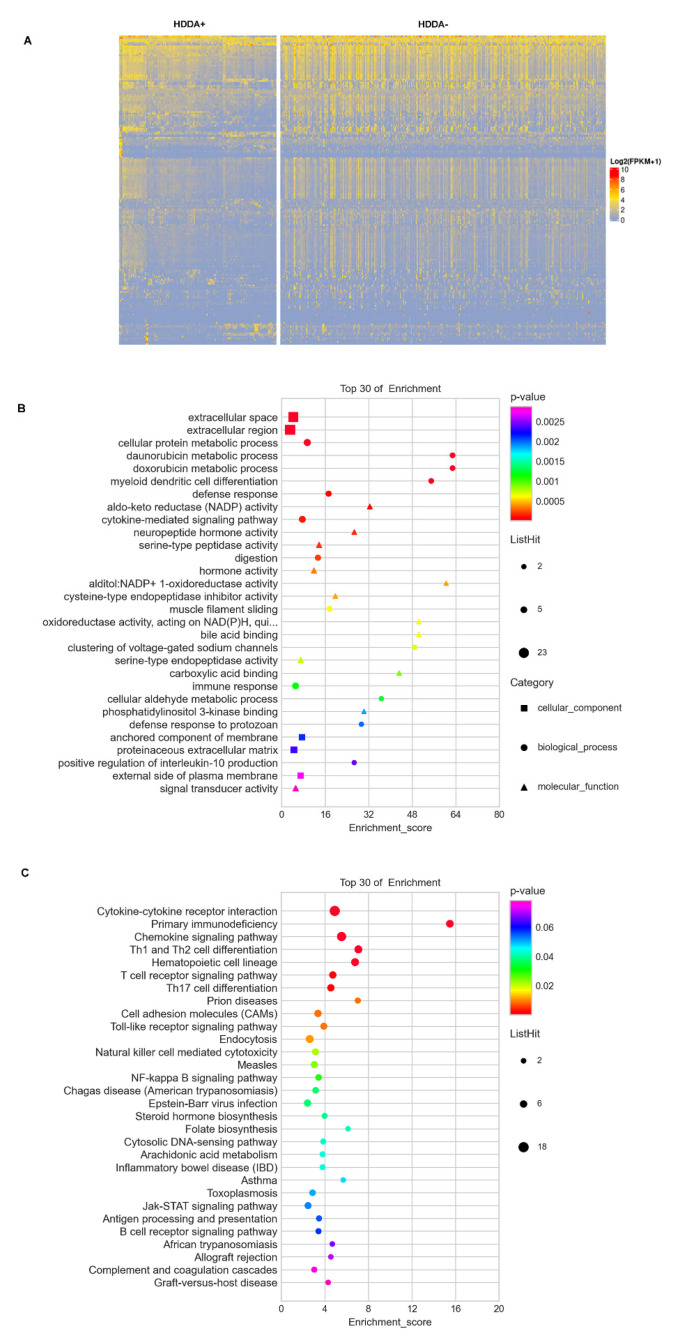
Differentially expressed genes (DEGs) and GO term/pathway enrichments in HDDA TC tumors. (**A**) The heatmap showing DEGs between tumors with and without HDDA based on the Table S3. There are 180 DEGs in tumors with HDDA (DNAmaa and DNAmad) compared to non-HDDA tumors, among which were 134 down- and 46 up-regulated genes, respectively. Scale: 0 to 10. (**B**) The enrichment of the GO terms in HDDA tumors. Shown are top 30 enriched GO terms including cellular components, biological processes and molecular function. (**C**) The KEGG analyses for the pathway enrichments in HDDA tumors. The top 30 enriched pathways are presented.

We then sought to determine whether DNAm age drift leads to alterations in gene expression in TC tumors. There were 180 DEGs in tumors with HDDA compared to non-HDDA tumors, among which were 134 down- and 46 up-regulated genes, respectively (Figure 4A and Table S3). To probe the biological functions of these DEGs, GO and KEGG pathway enrichment analyses were further carried out. Figure 4B,C showed top 30 altered GO terms and KEGG pathways, respectively. The GO-related DEGs were mainly involved in the metabolic activity, extracellular microenvironment components and immune response (Figure 4B), while the key enriched pathways for HDDA tumors were predominantly associated with inflammation/immunology regulations (Figure 4C).

In addition, we did not observe any overlapping genes between DEGs and DMPs identified in tumors with HDDA (Tables S2 and S3), which suggests that these DEGs may not be regulated by the DNA methylation.

### 3.7. Lower TDS in Tumors with HDDA

**Figure 5:**
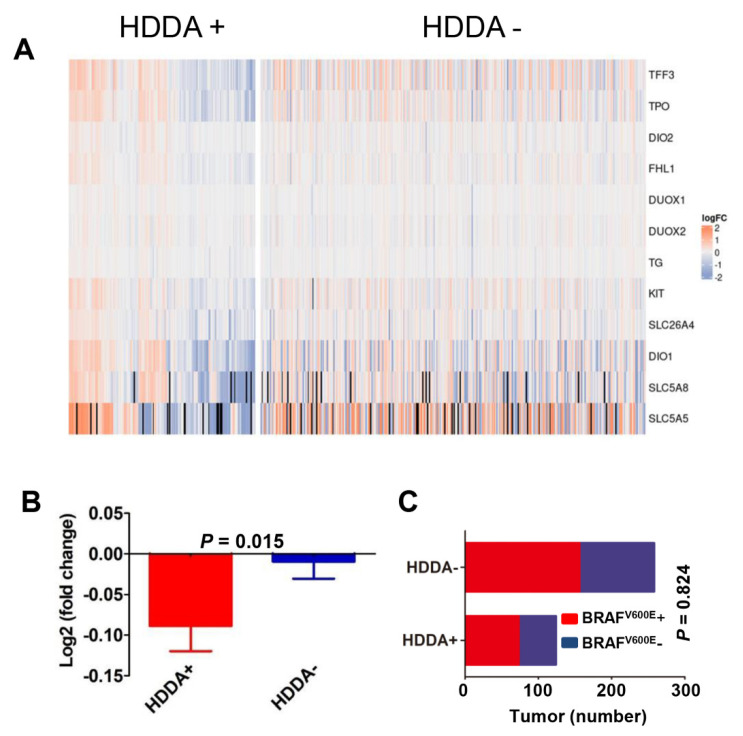
Lower thyroid differentiation score (TDS) in HDDA TC tumors. (**A**) The heatmap showing differential expression of 12 selected thyroid function genes (TG, TPO, SLC26A4, SLC5A5, SLC5A8, DIO1, DIO2, DUOX1, DUOX2, KIT, TFF3 and FHL1) in TC tumors with and without HDDA. Scale: −2 to +2. TDS calculation is described in Methods. (**B**) Significantly lower TDS in the tumor group with HDDA. Shown is the fold difference (Log2) between tumors with and without HDDA. (**C**) Lack of difference in HDDA between BRAF^V600E^+ and –PTCs. HDDA tumors (HDDA+) and tumors without HDDA (HDDA−) are analyzed for their BRAF^V600E^ mutations. The BRAF^V600E^ mutation has no effects on the presence of HDDA (*p* = 0.889). BRAF^V600E^+ and BRAF^V600E^−: with and without BRAF^V600E^ mutation, respectively.

The thyroid dedifferentiation plays a key role in the TC pathogenesis, and expression of thyroid metabolism/function genes has been established to determine TDS in PTC/FTC tumors [25]. To probe an association between TDS and DNAm age drift, we calculated TDS in each tumor using the expression panel of 12 selected genes (TG, TPO, SLC26A4, SLC5A5, SLC5A8, DIO1, DIO2, DUOX1, DUOX2, KIT, TFF3 and FHL1), as described [25], and significantly lower TDS values were observed in tumors with HDDA (Figure 5A,B, *p* = 0.015). Because the BRAF^V600E^ mutation is known to contribute to the loss of differentiation in PTC [25], we further examined whether the presence of BRAF^V600E^ was correlated with HDDA. There were 73 and 51 tumors with HDDA in 229 BRAF^V600E^+ and 153 BRAF^V600E^-PTCs, respectively, and no significant difference was observed (*p* = 0.824) (Figure 5C).

## 4. Discussion

Alterations in global DNA methylation play a critical role in both aging and cancer [12]. Therefore, insights into the epigenetic aging process should greatly contribute to our understanding of tumorigenesis. In TCs, DNA methylation aberration is widespread, and different histological subtypes were shown to exhibit specific methylation signatures. Those differentially methylated CpGs were strongly associated with distinct signaling pathways, tumor invasiveness, growth/proliferation and differentiation status [25,26,27]. Moreover, Bisarro dos Reis et al. identified that the prognostic classifier consisting of 21 CpG loci accurately predicted patient outcomes [27]. However, the relationship between DNAm age and clinical/pathological characteristics in TCs remains poorly understood. In the present study, our analyses of the TCGA TC cohort show that altered DNAm age are widespread and independently associated with poor patient outcomes.

Age is the strongest risk factor for the development and/or progression of many human malignancies. Indeed, recent genome-wide analyses of DNA methylation have shown particular features shared between aging and cancer [1,4,13,14]. Importantly, the accelerated DNAm age in normal cells derived from healthy individuals is associated with increased cancer risk and cancer-related mortality [8,9,11,17,18,21,28,29,30,31]. Devall et al. observed that higher DNAm age rates contributed to young age onset of colon cancer in African Americans [10]. Moreover, in patients with breast and colorectal cancer, their matched normal tissues frequently display significantly accelerated DNAm age [32,33,34,35]. However, our present results revealed that most of 54 NT samples (37/54) from TC patients displayed much younger DNAm age than their chronological age, which was contrary to the findings in other tissues above. The underlying mechanism is currently unclear. It is known that TCs can occur in both childhood and adulthood [36,37], with dramatical increase in women at reproductive age during the last decade [36], indicating that the TC pathogenesis is less strictly age-dependent. Therefore, TCs may still occur even if thyroid is at younger DNAm age. It should be pointed out, however, that the result obtained from 54 NT samples is not conclusive, and further investigations on larger cohorts are required to ascertain whether younger DNAm age in normal thyroid tissues is a general phenomenon in TC patients, and if so, why this happens.

Recent studies have shown that either DNAmaa or DNAmad in tumor tissues can be associated with poor patient outcomes dependent on cancer types. For instance, in cervical and breast cancer, and glioblastoma, DNAmad predicts significantly shorter overall and/or disease-free survival, while DNAmaa is associated with worse prognosis in colorectal cancer [16,18,19,38,39,40]. For these opposite observations, different explanations have been proposed. During malignant transformation, de-differentiation occurs and cancer cells usually acquire stem-like or immature phenotypes, which not only promotes proliferation, invasiveness or aggressiveness, but also reverses DNAm age [1,19]. It is thus conceivable that younger DNAm age and invasive phenotypes co-evolve in carcinogenesis to drive cancer progression, which might be the mechanism underlying the association between DNAmad and poor outcomes. On the other hand, DNAmaa may mimics senior age, because old age is a well-characterized factor for poor outcomes in many malignancies. However, our findings showed that high levels of either DNAmaa or DNAmad led to significantly shorter DFS in TC patients. This seemingly paradoxical scenario is difficult to explain by the proposals above. Likely, high DNAmaa and high DNAmad are both the indicator for the severest disturbance of DNA methylation, gene expression and signaling pathways in corresponding tumors. Indeed, these tumors display robust enrichments of metabolic and inflammatory pathways. All these may contribute to the progressive TCs.

Both PTCs and FTCs are in general considered as differentiated TCs, but dedifferentiation still occurs to various extents and they are very heterogenous in differentiation status [25]. Moreover, the loss of differentiation is associated with disease progression, poor outcomes and refractiveness to radioactive iodine treatment [25]. We found that tumors with HDDA exhibited significantly lower TDSs, which is likely an important contribution to poor patient outcomes. It is currently unclear how HDDA results in dedifferentiation of TCs. The loss of thyroid differentiation in PTCs has been attributable to the presence of BRAF^V600E^ [25], but we did not observe its association with HDDA. Therefore, HDDA-related TDS decline is likely independent of BRAF mutation. Further investigations are required to elucidate this issue.

The present study has limitations. In NT specimens from 54 TC patients, DNAm age was highly correlated with chronological age, but drifted to younger than chronological age in most of them, which is unexpected. However, the analyzed cohort is small, and further investigations on larger numbers of NTs are required to validate this finding for a solid conclusion. In addition, this TCGA cohort only contains PTC and FTC patients who in general have good prognosis. It remains to be defined whether HDAA contributes to more aggressive anaplastic TCs or poorly differentiated TCs.

## 5. Conclusions

The results presented herein reveal that most NT tissues from 54 TC patients exhibit younger DNAm age than chronological age, which is contrast to patients with breast and colorectal cancer, and other malignancies whose DNAm age in their corresponding NTs is much older. On the other hand, HDDA occurs in approximately 1/3 of TCs including PTCs and FTCs. In multi-variate analyses including age, extrathyroidal extension, TNM stage, and TDS, the presence of HDDA is the only variable to predict shorter patient DFS, while age > 55 is independently associated with shorter OS. HDDA tumors are characterized by loss of differentiation, and significantly altered metabolic activities, extracellular microenvironment components and inflammatory/immunological responses. All these changes are associated with aggressive behaviors of TCs. Likely, HDDA is one part of the integrated oncogenic program in TC pathogenesis and progression, and serves as a useful biomarker for TC prognostication.

## Figures and Tables

**Table 1:** Clinic-pathological characteristics for the TCGA TC patients.

Variable (Number)	*n* (%)
**Age at diagnosis (*n* = 508)**	
Median (Min–Max) (years)	46 (15–89)
<55	342 (67.3)
≥55	166 (32.7)
**Gender (*n* = 507)**	
Female	371 (73.2)
Male	136 (26.8)
**Histology (*n* = 508)**	
PTC	399 (78.5)
FTC	107 (21.1)
Other/unknown	2 (0.4)
**Tumor size (*n* = 502)**	
<4 cm	390 (77.7)
≥4 cm	112 (22.3)
**N** **stage (*n* = 458)**	
N0	233 (50.9)
N1	225 (49.1)
**TNM stage (*n* = 506)**	
I & II	338 (66.8)
III & IV	168 (33.2)
**Extrathyroidal extension (*n* = 489)**	
No	336 (68.7)
Yes	153 (31.3)

**Table 2:** Chronological and DNAm age in non-tumorous thyroid tissues and tumors from patients with thyroid carcinoma.

Specimen	Number	Chronological Age (Year)/Range (Median)	DNAm Age (Year)/Range (Median)
NT ^1^	54	15–81 (42)	12–66 (38)
TC Tumor	502		
All	502	15–89 (46)	8–121 (51)
PTC ^2^	396	15–89 (46)	15–107 (51)
FTC ^3^	105	16–83 (46)	8–121 (52)

^1^ NT: Non-tumorous thyroid tissue; ^2^ PTC: Papillary thyroid carcinoma; ^3^ FTC: Follicular thyroid carcinoma.

## Data Availability

In the present study, all the results were obtained from the analysis of the TCGA cohort of thyroid carcinoma and shown in the article and supplementary materials.

## Supplementary Materials

The following are available online at https://www.mdpi.com/article/10.3390/cancers13194827/s1, Figure S1: The association between highly drifted DNAm age (HDDA) and survival in papillary (A) PTC. (B) FTC. Left: overall survival. Right: disease-free survival, Table S1: DNA methylation age in paired NT and tumor specimens from 54 TC^1^ patients, Table S2: Differentially methylated CpGs, Table S3: Differentially expressed genes in tumors with highly drifted DNAm age (HDDA) down-regulated genes.
