# Supplementary material for: DNA Methylation Age Drift Is Associated with Poor Outcomes and De-Differentiation in Papillary and Follicular Thyroid Carcinomas
Source: Cancers (Basel). 2021 Sep 27;13(19):4827. doi: 10.3390/cancers13194827 (PMC8508076; doi:10.3390/cancers13194827)
Supplement: Supplementary file 1 [file cancers-13-04827-s001.zip › cancers-1346551-supplementary.pdf]

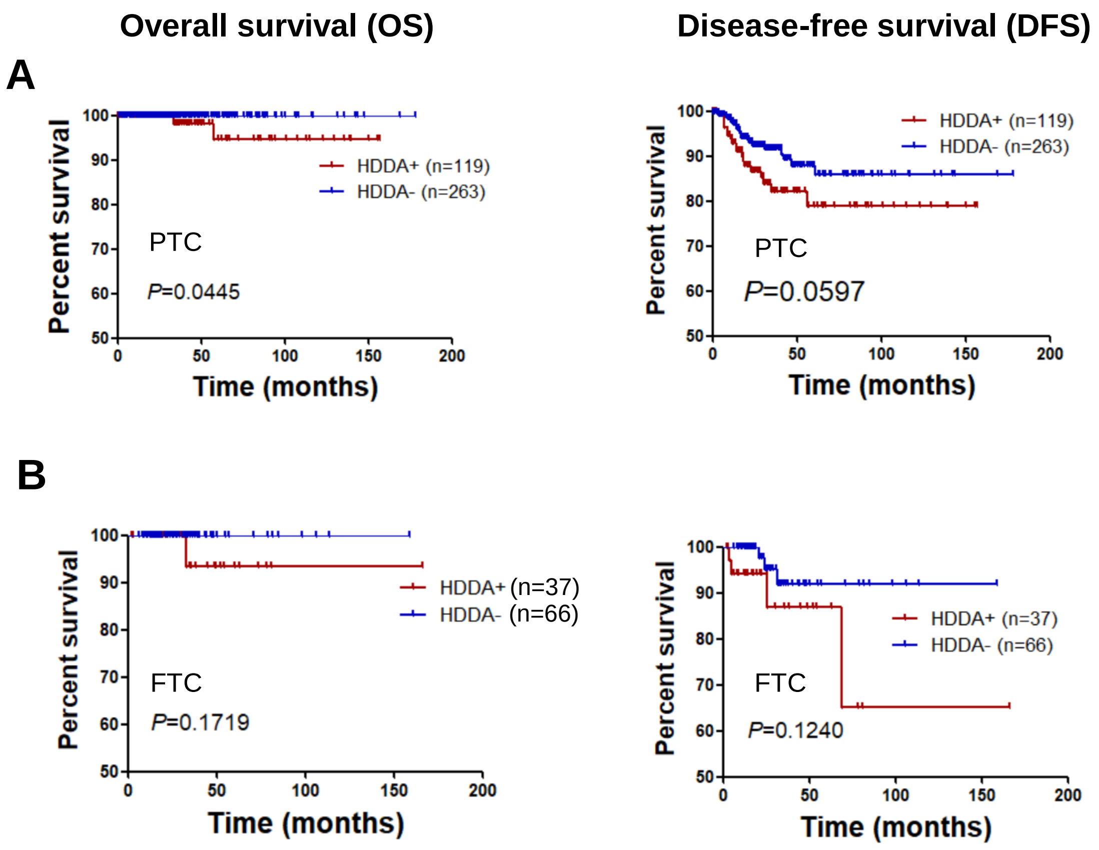

Figure S1. The association between highly drifted DNAm age (HDDA) and survival in papillary and follicular thyroid carcinoma (PTC and FTC). (A) PTC. (B) FTC. Left: Overall survival. Right: disease-free survival.

Table S1. DNA methylation age in paired NT and tumor specimens from 54 TC[1] patients.

Patient ID	Gender	Chronological age	DNAm age (NT)	Difference	DNAm age (T[2])	Difference
TCGA-EL-A3MY-11A	Male	81	60	-21	56	-25
TCGA-EL-A3ZO-11A	Female	79	64	-15	52	-27
TCGA-BJ-A2NA-11A	Male	77	63	-14	65	-12
TCGA-EM-A1CT-11A	Male	76	66	-10	64	-12
TCGA-EM-A1YC-11A	Female	71	65	-6	51	-20
TCGA-BJ-A290-11A	Male	70	58	-12	53	-17
TCGA-BJ-A3PR-11A	Female	69	59	-10	61	-8
TCGA-EL-A3H1-11A	Female	66	60	-6	90	24
TCGA-EL-A3MX-11A	Female	66	54	-12	54	-12
TCGA-ET-A3DW-11A	Male	64	54	-10	63	-1
TCGA-EL-A3T3-11A	Male	63	56	-7	56	-7
TCGA-EM-A3ST-11A	Female	62	60	-2	61	-1
TCGA-FY-A3TY-11A	Female	61	53	-8	60	-1
TCGA-EL-A3H2-11A	Male	58	60	2	60	2
TCGA-EL-A3MW-11A	Female	55	51	-4	68	13
TCGA-EL-A3T2-11A	Female	55	49	-6	64	9
TCGA-EM-A1CS-11A	Female	55	52	-3	64	9
TCGA-EL-A3N3-11A	Female	53	53	0	44	-9
TCGA-EM-A1YD-11A	Female	53	46	-7	55	2
TCGA-BJ-A3PU-11A	Male	52	47	-5	68	16
TCGA-EL-A3T7-11A	Female	47	51	4	40	-7
TCGA-EL-A3TB-11A	Female	47	43	-4	42	-5
TCGA-EM-A1YE-11A	Female	47	47	0	66	19
TCGA-EL-A3T0-11A	Female	45	43	-2	55	10
TCGA-ET-A3DP-11A	Female	43	37	-6	64	21
TCGA-BJ-A2N9-11A	Female	42	38	-4	35	-7
TCGA-EL-A3TA-11A	Female	42	36	-6	52	10
TCGA-EL-A3ZH-11A	Female	42	46	4	64	22
TCGA-EL-A3ZK-11A	Female	41	35	-6	53	12
TCGA-ET-A25J-11A	Female	40	35	-5	36	-4
TCGA-EM-A1CW-11A	Female	39	36	-3	43	4
TCGA-BJ-A28R-11A	Female	38	36	-2	50	12
TCGA-EL-A3T1-11A	Female	38	40	2	18	-20
TCGA-EL-A3H7-11A	Female	36	31	-5	57	21
TCGA-EL-A3T8-11A	Female	36	33	-3	80	44
TCGA-BJ-A28T-11A	Female	34	35	1	37	3
TCGA-EL-A3GZ-11A	Female	34	40	6	60	26
TCGA-EL-A3T6-11A	Female	34	36	2	30	-4
TCGA-EL-A3ZL-11A	Female	34	32	-2	77	43
TCGA-GE-A2C6-11A	Female	33	33	0	42	9
TCGA-BJ-A28W-11A	Female	32	28	-4	27	-5
TCGA-BJ-A28X-11A	Female	32	32	0	35	3
TCGA-EM-A1CV-11A	Female	32	30	-2	47	15
TCGA-EM-A1CU-11A	Female	31	32	1	55	24

TCGA-BJ-A2N7-11A	Female	30	30	0	47	17
TCGA-BJ-A2N8-11A	Female	30	33	3	47	17
TCGA-H2-A3RI-11A	Female	29	34	5	60	31
TCGA-ET-A25N-11A	Female	28	31	3	42	14
TCGA-H2-A2K9-11A	Female	25	27	2	38	13
TCGA-EL-A3N2-11A	Female	24	28	4	67	43
TCGA-DO-A1JZ-11A	Female	23	20	-3	19	-4
TCGA-EL-A3ZP-11A	Female	19	15	-4	15	-4
TCGA-E8-A2JQ-11A	Female	18	17	-1	13	-5
TCGA-EL-A3ZG-11A	Female	15	12	-3	9	-6

1 TC：Thyroid carcinoma; 2 T: Tumor

Table S2. Differentially methylated CpGs.

Probe ID	Gene name	Highly drifted	Others	deltaBeta	t	P.Value	adj.P.Value
cg00120783		0.510199367	0.571762006	-0.061562639	-3.4507866	0.000607681	0.044943828
cg00164949	C1QL1	0.483653165	0.385695137	0.097958028	4.481999037	9.23E-06	0.024800028
cg00405086	MED23	0.274141772	0.199110942	0.07503083	3.608691943	0.000339644	0.036424283
cg00498604	TLL1	0.230008861	0.156543161	0.0734657	4.041601317	6.17E-05	0.026295696
cg00622702	IFNAR1	0.660696203	0.597033435	0.063662768	3.487323698	0.000532172	0.042566653
cg00950086		0.495886076	0.558664134	-0.062778058	-3.50681826	0.000495564	0.041544402
cg00983904	IFFO1	0.585862025	0.502521884	0.083340141	4.384347149	1.43E-05	0.025301671
cg01047631		0.382440506	0.312550456	0.06989005	3.784936761	0.00017296	0.030748962
cg01254505	BST2	0.4225	0.36231459	0.06018541	3.615682719	0.000330842	0.036215027
cg01310473	CCDC146;FGL2	0.593170886	0.518524924	0.074645962	3.681887819	0.000257468	0.033787312
cg01329005	BST2	0.645282911	0.585245593	0.060037319	3.400050143	0.00072922	0.048096002
cg01355957	IFIT5	0.219756329	0.158832219	0.06092411	3.540558195	0.000437716	0.039656314
cg01586116	NCKAP5	0.415	0.333440426	0.081559574	3.644435072	0.000296845	0.035204041
cg01742627		0.213772785	0.135451368	0.078321417	4.168671191	3.63E-05	0.025670665
cg01743020	MYT1L	0.456087342	0.527808511	-0.071721169	-3.70792938	0.000233043	0.033033192
cg01790646	HDAC4	0.235941139	0.168077812	0.067863328	4.102574314	4.79E-05	0.025670665
cg01807407	NRN1	0.265241139	0.200378419	0.06486272	3.776162739	0.000178983	0.030872582
cg01817393	GNAS	0.240491772	0.319144377	-0.078652605	-4.0456326	6.07E-05	0.026151927
cg01821058	MMP2	0.305377215	0.239255623	0.066121592	3.594064743	0.000358775	0.037038886
cg02150820	HRNBP3	0.422081013	0.492413982	-0.070332969	-4.30413773	2.03E-05	0.025501775
cg02162404		0.536918354	0.464731307	0.072187047	3.337351335	0.000910681	0.05283613
cg02181920	TAP1	0.745139241	0.672965957	0.072173283	4.479905034	9.32E-06	0.024800028
cg02216731		0.244431013	0.313491185	-0.069060173	-4.30452432	2.02E-05	0.025501775
cg02318139	ST8SIA4	0.326981013	0.265249544	0.061731469	3.525473593	0.00046275	0.040363794
cg02333960	COX19	0.354348734	0.414758663	-0.060409928	-3.73457572	0.000210321	0.032069602
cg02371119	PLD1	0.456355696	0.393802432	0.062553265	3.171222019	0.001613933	0.0681072
cg02391713		0.574418354	0.470261094	0.10415726	4.012592701	6.95E-05	0.026323565
cg02621151		0.484289241	0.40946079	0.07482845	3.44418644	0.000622345	0.045385063
cg02685896	PROCA1	0.451018987	0.340843465	0.110175522	5.418258471	9.49E-08	0.017693885
cg02733847	LY6G5C	0.240270253	0.171723708	0.068546545	3.985866754	7.76E-05	0.026628071
cg02858512	SMG6	0.600276582	0.518559574	0.081717008	3.869431253	0.000123968	0.028508303
cg02933909	PTCHD3	0.747074684	0.686439818	0.060634866	3.673019588	0.000266321	0.034181668
cg02948656		0.527367089	0.587821884	-0.060454796	-3.49917696	0.000509623	0.04183981
cg02978168		0.642083544	0.572809119	0.069274426	4.444640713	1.09E-05	0.024800028
cg02989244		0.317817722	0.251279635	0.066538086	3.111631024	0.001970073	0.074401961
cg03036557	GPC5	0.307205696	0.242732827	0.064472869	3.438006264	0.000636375	0.045770854
cg03056526	EVX1	0.371786076	0.300571125	0.071214951	5.062744879	5.88E-07	0.019674773
cg03271907	MGMT	0.702586709	0.766686018	-0.064099309	-3.79115599	0.000168807	0.030607565
cg03397307	EFCAB4B	0.153259494	0.092360486	0.060899007	3.86121535	0.000128083	0.028612895
cg03487935		0.576434177	0.516016717	0.06041746	3.707566847	0.000233368	0.033033192
cg03627261	ARL15	0.332126582	0.393500912	-0.06137433	-3.52934876	0.000456194	0.040196146
cg03723247		0.301784177	0.226651064	0.075133113	3.405660409	0.000714744	0.047714092
cg03850256		0.441677215	0.359943769	0.081733446	3.44305586	0.00062489	0.04540625
cg03852144	GLRX	0.701811392	0.628889058	0.072922335	4.704769149	3.32E-06	0.024800028

cg03884238	C9orf152	0.741977848	0.804480243	-0.062502395	-3.38304003	0.000774797	0.049146619
cg04015907	FAM110A	0.275709494	0.18721155	0.088497944	4.515668796	7.93E-06	0.024800028
cg04046669	HORMAD2	0.517222785	0.44425076	0.072972025	3.786267372	0.000172064	0.030734493
cg04122873		0.344572152	0.268127052	0.0764451	3.868241073	0.000124556	0.028524561
cg04154027	CMYA5	0.184513291	0.244711854	-0.060198563	-4.11918379	4.47E-05	0.025670665
cg04254487	TBPL1	0.777731646	0.715594833	0.062136813	3.398784462	0.000732523	0.048184639
cg04257105	GNAS	0.292449367	0.361157143	-0.068707776	-4.83411987	1.80E-06	0.024800028
cg04373359	TMEM30B	0.512310759	0.450810638	0.061500121	3.652724692	0.000287671	0.035052196
cg04396998	PPP1R15A	0.366539241	0.290254407	0.076284833	3.755864791	0.000193684	0.031272641
cg04486778		0.304639873	0.234889362	0.069750512	3.844195577	0.000137021	0.02891693
cg04522045		0.369321519	0.297024316	0.072297203	3.442114485	0.000627016	0.045476947
cg05002602	VTI1B	0.287225949	0.347700304	-0.060474355	-3.40459629	0.000717469	0.047768669
cg05050657		0.349698734	0.280742553	0.068956181	3.392987452	0.000747831	0.048477979
cg05127899		0.325705063	0.247837386	0.077867677	3.726175829	0.000217248	0.032372667
cg05137450		0.279753797	0.211384195	0.068369603	3.439070448	0.000633938	0.045725997
cg05213296	RPF2	0.601837975	0.514374772	0.087463203	3.661815437	0.000277916	0.034656138
cg05333568	C1orf65	0.225255063	0.288338602	-0.063083539	-3.95108886	8.93E-05	0.027297078
cg05404698		0.323468354	0.223088754	0.100379601	4.223717817	2.87E-05	0.025501775
cg05457768		0.328953797	0.267374164	0.061579633	3.821343299	0.000149952	0.029609264
cg05498007	KATNAL2	0.470867722	0.382251672	0.08861605	3.669167278	0.000270255	0.034343897
cg05529506	KATNAL2	0.420472785	0.330469301	0.090003484	4.074930366	5.38E-05	0.025670665
cg05589784		0.778929114	0.709792097	0.069137017	3.425117003	0.00066659	0.046393636
cg05656374	MX2	0.558903165	0.457758967	0.101144198	4.118519694	4.48E-05	0.025670665
cg05810170		0.592663291	0.51412766	0.078535632	3.669815876	0.000269589	0.034314523
cg05895665		0.489796203	0.403085714	0.086710488	3.76517787	0.000186803	0.031077892
cg05905176	TNFAIP8L3	0.148966456	0.072749848	0.076216608	4.753896444	2.63E-06	0.024800028
cg05961294		0.616989873	0.548442249	0.068547624	3.622505795	0.000322459	0.036018892
cg06085204	XAF1	0.486503165	0.401908511	0.084594654	4.366515579	1.54E-05	0.025301671
cg06127263	DTHD1	0.732737975	0.669385714	0.06335226	3.605619712	0.000343581	0.036547752
cg06192883	MYO5C	0.407660759	0.312575076	0.095085684	4.233750348	2.75E-05	0.025501775
cg06455149		0.58009557	0.513389058	0.066706512	3.511491539	0.000487146	0.041294231
cg06523224	BNC1	0.311474051	0.245122492	0.066351558	3.472426625	0.000561833	0.043492388
cg06571387	HOXD12	0.399324684	0.329262006	0.070062677	5.169383146	3.44E-07	0.019674773
cg06644669	KIAA1949	0.644908228	0.582427964	0.062480264	4.483522958	9.17E-06	0.024800028
cg06666025	CMTM2	0.475151266	0.388497264	0.086654001	3.894619573	0.000112118	0.028288787
cg06874323		0.306722152	0.237434347	0.069287805	4.301248272	2.05E-05	0.025501775
cg07077965		0.652320253	0.565358055	0.086962198	4.075475699	5.36E-05	0.025670665
cg07095230	TBX2	0.403685443	0.322442553	0.08124289	3.401241591	0.000726123	0.048044662
cg07105596	GNAS	0.235703797	0.31571155	-0.080007753	-4.62763779	4.75E-06	0.024800028
cg07124687		0.279317089	0.191942857	0.087374231	4.176739407	3.51E-05	0.025670665
cg07169660	LOC100130581	0.499699367	0.59183769	-0.092138323	-4.18380302	3.40E-05	0.025670665
cg07337434	C9orf135	0.601156962	0.534817629	0.066339333	3.530478634	0.000454299	0.040185786
cg07385423	FCRLB	0.453925949	0.362183283	0.091742667	3.871126818	0.000123134	0.028508303
cg07523753	FLJ42875	0.489039873	0.426531915	0.062507959	3.46574755	0.000575627	0.044013509
cg07673538	FEZ2	0.467856329	0.385155319	0.08270101	4.034362313	6.36E-05	0.026295696
cg07696842	CHST11	0.266436076	0.199588146	0.06684793	3.599652904	0.00035135	0.036816236
cg07851675		0.441127215	0.377984802	0.063142413	3.833691845	0.000142828	0.029270182

cg07905965		0.35701962	0.296337386	0.060682234	3.687218081	0.000252281	0.033641446
cg08101264	ACOT8;ZSWIM3	0.288520886	0.222941641	0.065579245	4.543592013	6.99E-06	0.024800028
cg08167706	AKR1B1	0.239201899	0.178350456	0.060851443	4.35936167	1.59E-05	0.025301671
cg08215831	CRH	0.410120253	0.476834347	-0.066714093	-3.90598806	0.000107128	0.02814167
cg08241307		0.395884177	0.329515502	0.066368676	4.307030919	2.00E-05	0.025501775
cg08244085		0.521421519	0.441960486	0.079461033	3.92092579	0.000100891	0.027847218
cg08284263		0.737710759	0.661110942	0.076599817	3.427528356	0.000660838	0.046357123
cg08343644	GPR56	0.235252532	0.296484195	-0.061231663	-4.10361245	4.77E-05	0.025670665
cg08520746		0.355756329	0.293368693	0.062387636	4.534736859	7.27E-06	0.024800028
cg08793894	NEURL4	0.59926962	0.528870821	0.0703988	4.048564535	6.00E-05	0.026151927
cg08875705	IFFO1	0.719925316	0.650051368	0.069873949	3.817848139	0.000152029	0.029695103
cg09081997		0.646193038	0.565679331	0.080513707	3.605519561	0.00034371	0.036547752
cg09084462	NCRNA00202	0.249620886	0.18302614	0.066594746	4.784067945	2.28E-06	0.024800028
cg09147131	SLC16A3	0.25129557	0.178097872	0.073197697	3.895360548	0.000111786	0.028288787
cg09180171		0.397586709	0.336848328	0.060738381	3.848274124	0.000134827	0.028869047
cg09180564	OLFM3	0.663737975	0.59608693	0.067651045	3.57895375	0.000379598	0.037790253
cg09223687		0.235294304	0.296664134	-0.06136983	-4.56452725	6.35E-06	0.024800028
cg09229918	SYNGR3	0.270913291	0.196584195	0.074329097	4.072185248	5.44E-05	0.025767059
cg09230905	C11orf91	0.195573418	0.130867173	0.064706244	4.210365987	3.04E-05	0.025501775
cg09326440	HLA-E	0.491608228	0.426316717	0.065291511	3.545624573	0.000429596	0.039387861
cg09359103	KCNN3	0.379163291	0.317533435	0.061629856	3.478185646	0.000550188	0.04305808
cg09384411		0.525420886	0.465085714	0.060335172	3.386516199	0.000765272	0.04892522
cg09531959	LST1	0.572998734	0.510944681	0.062054053	3.916129604	0.000102855	0.027847218
cg09563922		0.272177215	0.204764742	0.067412474	4.330678404	1.81E-05	0.025501775
cg09577367	IL12RB1	0.720986709	0.646900304	0.074086405	4.852989051	1.64E-06	0.024800028
cg09596958	AGAP2	0.1901	0.252453495	-0.062353495	-4.11342271	4.58E-05	0.025670665
cg09620840	HLA-E	0.298451266	0.236825532	0.061625734	4.868136939	1.53E-06	0.024800028
cg09655952	PROK2	0.26196962	0.200255623	0.061713997	3.94294421	9.23E-05	0.027297078
cg09761080	LST1	0.580382911	0.50246535	0.077917562	4.462828577	1.01E-05	0.024800028
cg09854734	CMTM2	0.645772152	0.56502462	0.080747532	4.143025266	4.04E-05	0.025670665
cg10012059	STAP2	0.221713924	0.156653191	0.065060733	3.571753231	0.000389914	0.038203167
cg10123662	ARHGEF10L	0.612510759	0.549008511	0.063502249	3.958611209	8.66E-05	0.027105123
cg10195962		0.433709494	0.356413678	0.077295816	3.612704645	0.000334565	0.036323533
cg10302550	GNAS	0.313789873	0.385277204	-0.07148733	-4.88962322	1.38E-06	0.024800028
cg10356187		0.35613038	0.283186322	0.072944058	4.35588311	1.62E-05	0.025301671
cg10381888	HLA-L	0.717729114	0.654165653	0.06356346	3.600152186	0.000350693	0.036816236
cg10513595		0.678313924	0.591176596	0.087137328	3.822681762	0.000149164	0.029609264
cg10548038	GPC5	0.220874684	0.152136474	0.068738209	3.960906476	8.58E-05	0.027105123
cg10692140		0.556536076	0.489602736	0.06693334	3.445276201	0.000619902	0.045344118
cg10766585	SOD3	0.667990506	0.607989058	0.060001449	3.818755594	0.000151487	0.029695103
cg10806586	C14orf28	0.537866456	0.615613982	-0.077747526	-3.57294753	0.000388185	0.038141283
cg10861751	RGS1	0.514961392	0.448658967	0.066302426	3.977004834	8.04E-05	0.026794854
cg10919522	C14orf43	0.442787342	0.372664438	0.070122904	3.659186745	0.000280704	0.034766732
cg10949007	GLRX	0.583860759	0.505144985	0.078715775	3.874282552	0.000121597	0.028508303
cg10984625	LRRFIP1	0.760929114	0.695487538	0.065441576	4.832772554	1.81E-06	0.024800028
cg10995755	TPRKB	0.431518354	0.353037386	0.078480968	3.458366958	0.000591237	0.04447844
cg11117438		0.262886076	0.338441945	-0.075555869	-4.30552689	2.02E-05	0.025501775

cg11139090	UNC5A	0.468582278	0.380881459	0.08770082	3.550854926	0.000421362	0.039172133
cg11155687		0.592691772	0.527774772	0.064917	3.388636413	0.000759517	0.04876842
cg11224765	ODF3B	0.537467089	0.461960182	0.075506906	3.764924911	0.000186987	0.031077892
cg11236452	PCDHGA2	0.531956962	0.43881155	0.093145412	4.089672079	5.06E-05	0.025670665
cg11238542		0.365937975	0.279005167	0.086932808	3.711396415	0.000229961	0.032935277
cg11316887	SVIL	0.778107595	0.71372614	0.064381455	4.394035035	1.37E-05	0.025301671
cg11354682	C19orf38	0.779886709	0.690042857	0.089843852	3.879305056	0.000119188	0.028506687
cg11374933		0.708393038	0.64103465	0.067358388	3.498363217	0.000511142	0.041918381
cg11460496		0.503827215	0.416344377	0.087482838	3.822863232	0.000149058	0.029609264
cg11539674	SND1	0.367546203	0.294198176	0.073348026	3.878177148	0.000119725	0.028506687
cg11549953		0.296287975	0.221199392	0.075088583	3.647045914	0.000293926	0.035120189
cg11660826	ULBP1	0.188708861	0.121980851	0.06672801	4.458040896	1.03E-05	0.024800028
cg11738486	TRIP10	0.283627215	0.346155015	-0.0625278	-4.3229084	1.87E-05	0.025501775
cg11806439		0.258360127	0.321269301	-0.062909174	-4.38982094	1.39E-05	0.025301671
cg11931731	KATNAL2	0.443970253	0.347929483	0.09604077	3.447306595	0.000615372	0.045195698
cg11985341	TMEM45B	0.391581013	0.45248845	-0.060907437	-3.70343492	0.000237097	0.033232589
cg12111714	ATP8A2	0.430641139	0.356874164	0.073766975	3.894188511	0.000112311	0.028288787
cg12236164		0.494967089	0.419383891	0.075583198	3.60830916	0.000340132	0.036464992
cg12242502	GSTA1	0.632859494	0.695272644	-0.062413151	-3.95030531	8.96E-05	0.027297078
cg12302982		0.619967722	0.536143769	0.083823953	3.579718127	0.000378518	0.037778643
cg12434681	HOXA3	0.347975949	0.420989666	-0.073013716	-3.64118323	0.000300519	0.03525501
cg12442246		0.327488608	0.239721581	0.087767027	3.715975362	0.00022595	0.032804288
cg12505153	CFD	0.595858861	0.534344985	0.061513876	3.97016627	8.27E-05	0.026981582
cg12578486	UBAC2	0.619435443	0.556659271	0.062776173	3.614558515	0.000332243	0.036257899
cg12666727	HIVEP3	0.222286076	0.14180304	0.080483036	3.871555959	0.000122924	0.028508303
cg12671030		0.290927848	0.228412766	0.062515082	3.549263984	0.00042385	0.03926202
cg13231131	DUSP27	0.608210759	0.543384195	0.064826565	3.372591032	0.000804095	0.049976017
cg13326508	MPP2	0.340094937	0.267105167	0.07298977	3.567088763	0.000396736	0.038453734
cg13360395		0.380979114	0.317990274	0.06298884	3.619229603	0.000326459	0.036086161
cg13522462	ZNF284	0.198939873	0.265664134	-0.06672426	-3.98416568	7.81E-05	0.026725016
cg13571293		0.718982911	0.651858055	0.067124857	3.896524621	0.000111267	0.028269738
cg13656173		0.61691519	0.552626748	0.064288442	3.627936409	0.000315929	0.03580781
cg13967811	EPB41L4A	0.63251519	0.560632523	0.071882667	3.992160216	7.56E-05	0.026554974
cg13996522	TRAF3	0.271767722	0.33942766	-0.067659938	-3.87530516	0.000121103	0.028508303
cg14195925	HRNBP3	0.153486709	0.216992401	-0.063505692	-4.84708854	1.69E-06	0.024800028
cg14302936	GLIPR2	0.735491772	0.675375988	0.060115784	3.610471609	0.000337382	0.036350891
cg14440974		0.326886709	0.411366261	-0.084479553	-4.14946181	3.93E-05	0.025670665
cg14514569		0.357658861	0.292585714	0.065073146	3.781439831	0.000175337	0.030814643
cg14676825	PROCA1	0.438808861	0.327144377	0.111664484	5.149361175	3.80E-07	0.019674773
cg14775960		0.416322152	0.484861398	-0.068539246	-3.55967281	0.000407813	0.038703645
cg15215155	FLNC	0.451155696	0.385086626	0.06606907	3.42288697	0.000671952	0.046618903
cg15281283	MX2	0.572279747	0.462906079	0.109373668	4.474017739	9.57E-06	0.024800028
cg15426869		0.476701899	0.40142462	0.075277279	3.664477947	0.000275118	0.034548078
cg15448445	CMKLR1	0.159958861	0.093982371	0.06597649	4.876355608	1.47E-06	0.024800028
cg15465279		0.29891962	0.367045593	-0.068125972	-5.55617128	4.55E-08	0.016971569
cg15554005		0.765037975	0.700010334	0.06502764	4.20242207	3.14E-05	0.02563568
cg15560495		0.415187975	0.353657143	0.061530832	4.163367447	3.71E-05	0.025670665

cg15852963	SOX2OT	0.393448734	0.328385714	0.06506302	3.754610115	0.000194629	0.031304184
cg15896259	ENTPD4	0.699724684	0.634858663	0.064866021	3.546907041	0.000427563	0.039327153
cg16206813		0.578896835	0.505421277	0.073475559	3.408563023	0.00070736	0.047473413
cg16271437		0.703789241	0.643448936	0.060340304	3.871519707	0.000122942	0.028508303
cg16385335	IL18BP	0.435692405	0.368195137	0.067497268	3.565724378	0.000398752	0.038505326
cg16426479		0.517796203	0.441820669	0.075975534	3.378947339	0.000786152	0.049446425
cg16606773	RIN2	0.158287342	0.226886322	-0.06859898	-4.36007655	1.59E-05	0.025301671
cg16626067	CMTM2	0.550260759	0.483438906	0.066821854	2.927468757	0.003577603	0.097356431
cg16651780	C16orf61	0.496967089	0.406442249	0.090524839	3.908364587	0.000106112	0.028123701
cg16676676	OBSCN	0.673781646	0.745986626	-0.072204981	-3.53012605	0.000454889	0.040185786
cg16677191	GLRX	0.667313924	0.594948024	0.0723659	4.059175637	5.74E-05	0.026062136
cg16795307	STARD13	0.687949367	0.623565653	0.064383714	4.49172674	8.84E-06	0.024800028
cg17084763		0.598839241	0.526432219	0.072407022	3.878488549	0.000119577	0.028506687
cg17096289	HLA-C	0.519793671	0.429802432	0.089991239	4.122096209	4.41E-05	0.025670665
cg17124224	BNC1	0.202115823	0.141172036	0.060943786	3.88821109	0.000115027	0.028365033
cg17239876	KIAA1274	0.186150633	0.247444985	-0.061294352	-4.465657	9.94E-06	0.024800028
cg17386213	NR2E1	0.202305063	0.134131915	0.068173148	3.99012504	7.62E-05	0.026595062
cg17414107	GNAS	0.357822785	0.43548845	-0.077665665	-4.55697367	6.57E-06	0.024800028
cg17493815	GUCY1B3	0.551536709	0.463491793	0.088044916	5.299551248	1.76E-07	0.019674773
cg17608381	HLA-A	0.79366962	0.732971125	0.060698496	4.139685666	4.10E-05	0.025670665
cg17666539	EVI5L	0.328614557	0.263568085	0.065046472	3.248397989	0.001240894	0.060366004
cg17880320	PEX19	0.519920253	0.584121884	-0.064201631	-3.57269333	0.000388552	0.038152629
cg17941572		0.370129114	0.284491489	0.085637625	3.877826291	0.000119893	0.028506687
cg18339788		0.193217089	0.126441337	0.066775751	3.564771798	0.000400166	0.038505326
cg18537730	IZUMO1	0.454603797	0.360075988	0.09452781	3.560057878	0.000407231	0.038686619
cg18579761		0.314817722	0.384917325	-0.070099604	-3.61406708	0.000332857	0.036276015
cg18634709		0.370422152	0.43175228	-0.061330128	-3.39116444	0.000752707	0.048619085
cg18640030	CRH	0.365631013	0.445013678	-0.079382665	-3.70297753	0.000237513	0.033247372
cg18761422		0.643932911	0.571121884	0.072811027	3.848597554	0.000134654	0.028869047
cg18792131		0.372051266	0.45511307	-0.083061804	-3.38221071	0.000777085	0.049224811
cg18882687	ARF1	0.639018354	0.572561702	0.066456652	3.373087301	0.00080268	0.049946955
cg19029181	ZIC1	0.416732911	0.346429179	0.070303732	3.6092515	0.000338931	0.036388563
cg19094438	BCAR1	0.601586709	0.533890578	0.067696131	4.039157885	6.23E-05	0.026295696
cg19167683	SLC43A3	0.363043038	0.284650152	0.078392886	3.533394938	0.000449441	0.039997659
cg19279342	CFD	0.299177215	0.222595441	0.076581774	3.716261412	0.000225701	0.032804288
cg19457763	ASAM	0.59035	0.526737082	0.063612918	3.779229989	0.000176856	0.030814643
cg19495714	SIVA1	0.80469557	0.731141337	0.073554232	3.680911219	0.000258429	0.033789497
cg19500334		0.652573418	0.589712462	0.062860956	3.478176444	0.000550206	0.04305808
cg19501909		0.629347468	0.563566261	0.065781207	3.838154787	0.000140333	0.029141242
cg19640589	GNAS	0.335725949	0.416308511	-0.080582561	-4.33788459	1.75E-05	0.025501775
cg19680672	MGMT	0.524105696	0.610570517	-0.086464821	-3.85314293	0.000132251	0.028857612
cg19697239	NINJ2	0.276079747	0.208796049	0.067283698	3.585877976	0.000369919	0.037441467
cg19713429	CAPZB	0.383163924	0.323044377	0.060119547	3.724080722	0.000219009	0.032444305
cg20164887	TYMP	0.502289873	0.417224012	0.085065861	3.82086335	0.000150236	0.029609264
cg20178976		0.684751266	0.623019757	0.061731509	3.633545583	0.000309315	0.035542082
cg20274304	PNOC	0.356878481	0.43993769	-0.083059209	-4.13670732	4.15E-05	0.025670665
cg20294304	HMGA1	0.330648734	0.269410942	0.061237792	3.462984148	0.000581426	0.044160092

cg20386316		0.272712658	0.20392766	0.068784999	3.478982504	0.000548594	0.043027329
cg20528093	PAX6	0.336629747	0.268144073	0.068485674	3.567034595	0.000396816	0.038453734
cg20594303		0.332572785	0.267002128	0.065570657	2.982504499	0.003002664	0.08993236
cg20673075	STK3	0.486462025	0.423698176	0.062763849	3.906270716	0.000107007	0.02814167
cg20773915	CHD5	0.725543671	0.789739514	-0.064195843	-3.3900787	0.000755625	0.048709187
cg20939662	LHX2	0.521250633	0.437221884	0.084028748	4.107273474	4.70E-05	0.025670665
cg21172319	NAT1	0.373748101	0.300084195	0.073663907	3.675470186	0.000263846	0.034030116
cg21176130	HLA-E	0.383825316	0.297459878	0.086365438	4.255849882	2.50E-05	0.025501775
cg21330896	ZNF395	0.722670253	0.655525532	0.067144721	3.478178169	0.000550203	0.04305808
cg21354781		0.691974684	0.617500608	0.074474076	3.644804602	0.00029643	0.035179767
cg21501525		0.666116456	0.606079635	0.06003682	4.625200493	4.80E-06	0.024800028
cg21870299		0.638151899	0.70144772	-0.063295822	-3.38937076	0.000757533	0.048720505
cg21884062	MIR548F5	0.691217089	0.627504863	0.063712225	3.727958561	0.00021576	0.032308619
cg21990700	LOC283314	0.383044304	0.317269909	0.065774395	3.69123044	0.000248441	0.033531083
cg22084642	DNAH1	0.348098101	0.265951368	0.082146733	3.560837555	0.000406054	0.038681088
cg22111078		0.279589873	0.215710942	0.063878931	3.434865392	0.000643618	0.045917241
cg22164298	ANK2	0.773514557	0.712171733	0.061342824	3.914743599	0.000103429	0.027847218
cg22254983		0.390047468	0.329372644	0.060674824	3.373476183	0.000801573	0.049903061
cg22272840	SOX7	0.619882278	0.552161702	0.067720576	3.382933047	0.000775092	0.049148605
cg22371492	PRRX1	0.713290506	0.647957447	0.06533306	3.625270692	0.000319119	0.035948124
cg22372182	C1orf65	0.193872785	0.277098784	-0.083225999	-4.20477248	3.11E-05	0.02563568
cg22413056	KIAA1949	0.534113924	0.46738693	0.066726994	4.045539017	6.07E-05	0.026151927
cg22541038		0.329727848	0.253248328	0.07647952	4.467946787	9.83E-06	0.024800028
cg22620090		0.544413924	0.454104255	0.090309669	3.461869878	0.00058378	0.044191496
cg22647546	FAM59B	0.437233544	0.372277508	0.064956037	3.498929197	0.000510085	0.041850107
cg22689909	GLRX	0.536518354	0.428396049	0.108122306	5.058824814	5.99E-07	0.019674773
cg22749810	RNF213	0.687637975	0.62518693	0.062451045	3.730481823	0.000213671	0.032173766
cg22821981	PARP15	0.59971962	0.528632219	0.071087401	4.032031812	6.42E-05	0.026295696
cg23133355		0.368753165	0.443496353	-0.074743188	-4.20295947	3.14E-05	0.02563568
cg23145794	RALGAPA2	0.609906329	0.529602736	0.080303594	4.284415433	2.21E-05	0.025501775
cg23224396	SAMD4A	0.71366519	0.653413678	0.060251512	3.744835478	0.000202142	0.03156071
cg23277659	MKNK1	0.565770886	0.629396049	-0.063625163	-3.85638702	0.000130561	0.028763285
cg23326313		0.300093038	0.234064742	0.066028296	3.975861181	8.08E-05	0.026794854
cg23426945		0.470412025	0.377343465	0.09306856	3.935948219	9.50E-05	0.027532481
cg23497383	LOC151174	0.257262658	0.193317021	0.063945637	3.416718685	0.000686992	0.047127319
cg23528751	CCDC50	0.403311392	0.342984802	0.06032659	3.52428625	0.000464776	0.04043271
cg23546474	PAX3	0.505341772	0.421852584	0.083489189	4.154726262	3.85E-05	0.025670665
cg23737737	IFFO1	0.661267089	0.585186018	0.07608107	4.48243672	9.21E-06	0.024800028
cg23747646	ZNF76	0.390013924	0.451551368	-0.061537444	-3.74718089	0.000200314	0.031514344
cg23762359	KCNQ1DN	0.401961392	0.336419149	0.065542243	3.892232744	0.000113193	0.028334548
cg23889684	APOL3	0.343252532	0.252094833	0.091157699	3.551399797	0.000420512	0.039141962
cg23971170	LMX1A	0.589788608	0.504451976	0.085336632	3.44836753	0.000613018	0.045114885
cg24043916		0.368557595	0.286230699	0.082326896	3.669877228	0.000269526	0.034314523
cg24244374		0.580482911	0.509882371	0.070600541	4.226686499	2.83E-05	0.025501775
cg24459409	CCDC61	0.363812025	0.294641337	0.069170688	3.504240838	0.000500265	0.041679073
cg24571822	CUBN	0.309617722	0.249090274	0.060527448	3.573559615	0.000387301	0.038118862
cg24590708	MYO5C	0.528839241	0.440453799	0.088385441	3.984834158	7.79E-05	0.02669955

cg24611608	EYA1	0.563303797	0.492127356	0.071176442	3.505020647	0.000498839	0.041664256
cg24683680	EVX2	0.285186709	0.21674924	0.068437469	3.503810823	0.000501054	0.041679073
cg24841318	PROCA1	0.489098101	0.396174772	0.092923329	4.807210841	2.04E-06	0.024800028
cg25162921	MTAP	0.223753797	0.284451672	-0.060697874	-3.77269149	0.00018142	0.030915543
cg25200152		0.263753165	0.196724012	0.067029152	3.838759022	0.000139998	0.029131738
cg25407540	C9	0.732656962	0.668556535	0.064100427	3.558507356	0.00040958	0.038758861
cg25597625	KIAA0319	0.312368987	0.388909422	-0.076540435	-3.77320735	0.000181056	0.030915543
cg25618573	KLC2	0.289866456	0.215340729	0.074525726	3.481156849	0.000544268	0.04286099
cg26118326	MCL1	0.464484177	0.390618845	0.073865332	3.739819518	0.000206102	0.031809241
cg26156321		0.717940506	0.655755319	0.062185187	3.817012727	0.000152529	0.029695103
cg26166688	COX17	0.367139241	0.303359878	0.063779362	4.209701569	3.05E-05	0.025501775
cg26188685	HDC	0.75081519	0.690793313	0.060021877	4.416544963	1.24E-05	0.025301671
cg26234900	TAP1	0.745009494	0.681224316	0.063785178	3.966881861	8.38E-05	0.027000047
cg26328291		0.257655696	0.321862614	-0.064206918	-3.55538064	0.000414356	0.038916141
cg26386409		0.437775316	0.365970517	0.0718048	3.512120759	0.000486022	0.041245888
cg26426745	C2CD4D	0.603376582	0.53596231	0.067414272	3.757076917	0.000192775	0.031237506
cg26513854		0.665131013	0.603538906	0.061592107	3.868873751	0.000124243	0.028512522
cg26534477	G6PC3	0.657062025	0.596683283	0.060378743	4.049594604	5.97E-05	0.026151927
cg26603179	C1orf65	0.224610127	0.28476079	-0.060150664	-4.05764911	5.77E-05	0.026068606
cg26689203	MYOZ2	0.682163924	0.60739696	0.074766964	3.796959054	0.000165017	0.030467819
cg26767059	KLHL29	0.552448101	0.61963769	-0.067189589	-3.77190658	0.000181976	0.030915543
cg26880239	CD247	0.507874051	0.439709119	0.068164932	3.778522605	0.000177344	0.030822682
cg26924440	IFFO2	0.586327848	0.521467781	0.064860067	3.497387421	0.000512969	0.041980531
cg26955987	EFCAB4B	0.148785443	0.086669301	0.062116142	3.98908507	7.65E-05	0.026595062
cg26978918		0.342139873	0.279506079	0.062633794	3.427717503	0.000660388	0.046357123
cg27071793		0.57685443	0.513547112	0.063307318	3.395430615	0.000741344	0.048300264
cg27507473		0.321575949	0.258257751	0.063318199	3.480166363	0.000546235	0.042897134
cg27513586	SNORD115-48	0.564850633	0.493283891	0.071566742	4.139040482	4.11E-05	0.025670665
cg27534671	THSD1	0.373741139	0.295797264	0.077943875	3.708739785	0.00023232	0.033014169
cg27625456	LGALS9	0.337967722	0.273123404	0.064844317	3.852227728	0.000132732	0.028869047

Table S3. Differentially expressed genes in tumors with highly drifted DNAm age (HDDA).

Down-regulated genes

gene name	DNAm age drift	Others	log2FC	Pvalue	Qvalue
MATN1	0.0462460572176512	1.99970346905352	-5.43431190047848	2.07814277418908e-40	4.3568263260874e-37
IGF2	2.15627997658626	22.1037850896855	-3.35767702195521	4.37484993191997e-30	5.64422946601245e-27
HP	0.123161289616379	1.01505189998598	-3.04293271075457	3.05456006214289e-21	2.13462839009419e-18
MSMP	4.72261462735139	20.1643398264954	-2.09414845392052	2.02821278229437e-17	1.06303702452004e-14
KRT6B	0.11863420296797	1.78667237168815	-3.91268319384599	3.19872270841511e-16	1.49024936848717e-13
KRT6A	0.435503318452817	4.25203850142891	-3.28739904322553	8.77502147672271e-16	3.87301737388403e-13
ALK	0.32566206512111	1.2841848054869	-1.97940525219283	1.34551024586126e-15	5.78638406245772e-13
TCN1	0.146468353790846	1.29587947220915	-3.14527064936585	6.88948413701075e-15	2.51196582491183e-12
BCHE	0.383132964623263	1.3451517764067	-1.81185189921183	2.12179222087791e-12	6.03164392009563e-10
DPRX	0.0135474615396093	4.36102025382782	-8.33049932908969	4.70663665638418e-12	1.21445707693655e-09
IGF1	0.689765506796273	1.5789936340271	-1.1948274640921	1.26840475638276e-11	3.08314269189153e-09
KRT5	0.394864086444318	2.15872062213189	-2.45074848103182	4.09381129412445e-11	8.8027439775712e-09
CD79B	1.28140613193535	2.53918797638956	-0.986639402439997	1.27700455190462e-10	2.47246464377818e-08
S100A2	7.30425135220795	16.2917603123622	-1.15733417741495	1.28252101126104e-10	2.47246464377818e-08
BST2	19.0127725558547	31.6527611470165	-0.735362425205325	3.15127322609e-10	5.87257272755349e-08
NKG7	2.03804139781006	3.92005924943796	-0.943692103398921	3.8078640440564e-10	7.01818634581471e-08
TYMP	3.25425902277418	5.28779211185487	-0.70033637188289	3.95877458033244e-10	7.17008229568538e-08
CCL21	8.05404402248381	30.8144521597027	-1.93582187909644	6.09772366165878e-10	1.03304061872062e-07
PTPRCAP	9.655932891584	18.0503110136237	-0.902536140945524	8.89191123099527e-10	1.43399168429089e-07
CST7	3.77913938551283	7.27676296212065	-0.945239085895908	1.09118099441592e-09	1.72653656965508e-07
KRT14	0.598588741970606	1.88524440866685	-1.65511452033124	2.09245969101468e-09	3.10572866705293e-07
LAG3	0.500883485220174	1.05871842510548	-1.07977199374226	2.11410715530436e-09	3.11033379024252e-07
SFTPA2	2.25074643973019	9.03412346948936	-2.00498109366399	5.94324046993818e-09	7.55151736074267e-07
SLC5A5	1.26357827649044	5.53343183839577	-2.13065947902417	6.69545754480956e-09	8.3803144732497e-07
PDCD1	0.625675497408664	1.28424520350698	-1.03743417371951	7.67773550749142e-09	9.26409927565799e-07
CXCR3	0.826309169736672	1.46205648654269	-0.82324546800771	8.30350400738747e-09	9.80749078957061e-07
PIM2	5.60560277659796	9.81545572294409	-0.80818573635565	8.67125112478467e-09	1.01702254450971e-06
KRT17	3.35439217463965	9.96437710019378	-1.57072825148463	1.03634854008184e-08	1.1905231311132e-06
SLAMF7	1.14841545965586	2.62299848442683	-1.19157231705557	1.37135926452679e-08	1.53336250564289e-06
GZMA	3.94803511487323	7.5129051439079	-0.928236065703254	1.47123449949113e-08	1.63414205466657e-06
CD3D	2.22879272532127	4.51046421538175	-1.01701346861729	1.8044091512529e-08	1.96516560290998e-06
CCL5	10.2178414447259	19.1473535460218	-0.906054549630119	1.82673549230087e-08	1.97664565657227e-06
GZMM	0.641815135409164	1.21667627417925	-0.922715638543377	1.94234831296318e-08	2.07487920618824e-06
ACAP1	0.608800498572895	1.0016422049172	-0.718325812221102	4.21448273986941e-08	4.20379710932532e-06
DPT	3.05450937111354	7.71561181494927	-1.33683989430949	6.85091675055658e-08	6.41919417543771e-06
CHRDL1	0.756602596933855	1.82546126180192	-1.2706534202395	7.30941159905137e-08	6.8107472966272e-06
JSRP1	0.427696800419854	1.07988981927135	-1.33622380266458	8.09774328159619e-08	7.42160384256455e-06
SECTM1	0.877667054200859	1.43775028668866	-0.712067467716439	1.02470433851762e-07	8.8589387451637e-06
FCER1G	10.9139109291289	17.3564364865965	-0.669302598480641	1.12290642777299e-07	9.56009472416676e-06
GZMK	1.58757177983751	3.42346382195307	-1.10863494378299	1.34897319401422e-07	1.10244482043344e-05
CD3E	1.24093702784802	2.29302930935444	-0.885824888639655	1.35454237309775e-07	1.10244482043344e-05
FGL2	2.69095739679722	4.03949671505337	-0.586056006921778	1.56893432917921e-07	1.23540688117341e-05
CHGB	3.96211325248831	10.1367409984921	-1.35525187111983	1.59871192869238e-07	1.24714402176877e-05

C16orf54	0.71358313372947	1.27488160142912	-0.837209848664348	1.5913354799807e-07	1.24714402176877e-05
CCL4	1.46942461059272	2.48193193210664	-0.756212206718542	1.68319914997897e-07	1.30095005269343e-05
CXCL9	12.1852817165268	27.6963041198122	-1.18455386574012	1.82557375471787e-07	1.39175104609673e-05
GBP5	0.693786549732549	1.34500347377134	-0.955046122030545	1.91238697260384e-07	1.45133729884668e-05
CORO1A	3.93109073329537	6.2113230712373	-0.659970946112473	2.08656302061492e-07	1.55898574162614e-05
MYL4	3.73708651206443	6.73061546705898	-0.848824473178478	2.15569463030099e-07	1.59979249289417e-05
CCR7	0.596268258316211	1.09206701799307	-0.873027950678045	2.67048359913157e-07	1.93057547088943e-05
XCL2	0.758109246876989	1.46975996792537	-0.955102895109105	2.79174680184745e-07	2.00099048549511e-05
LCK	0.712590724738028	1.18887028732908	-0.738445707019658	2.86440581343452e-07	2.04433252352867e-05
CYTH4	1.08546731582505	1.67457838670748	-0.625481623858668	3.01034948715251e-07	2.13938905078482e-05
CD48	1.70510098386743	2.91755295181313	-0.774901655439739	3.6331489202195e-07	2.5284304435652e-05
SASH3	3.22821960368514	5.23351785034626	-0.697042295875958	3.91909133350298e-07	2.70497941751078e-05
IL2RB	2.43547171748509	3.98713608927611	-0.711151618744071	4.02881190735813e-07	2.75800952286574e-05
CD8A	1.86986389187489	3.11600694913645	-0.736765191219656	4.4473630049544e-07	2.95996715551965e-05
RUNX3	0.729847112488551	1.21667362504668	-0.7372760276324	4.76710120754447e-07	3.07166872862051e-05
STAT4	0.727146183082054	1.19319080919148	-0.714507437068976	4.75291543109267e-07	3.07166872862051e-05
BATF	1.05394519625869	1.86222488594219	-0.821227455397804	4.86065706101088e-07	3.11156260409444e-05
HSPA6	2.34635237434976	3.75427973485344	-0.67811645765894	6.60617951278784e-07	4.04343680516434e-05
LGALS2	0.715543382061852	1.48688123117898	-1.05517827003976	6.96033519148403e-07	4.19923531768238e-05
PRF1	1.16877784930225	1.79012951158355	-0.615063225264831	7.13227975590034e-07	4.25703188846835e-05
LIMD2	1.2522762113272	1.93936055782144	-0.631028239336705	8.08568908640487e-07	4.69161404099167e-05
HCST	6.42481963363026	9.7656157537953	-0.604055060032376	8.16068636153952e-07	4.70347187820415e-05
IL2RG	2.40472914799067	4.2287479688744	-0.814356171088695	9.22993107638947e-07	5.16014680044014e-05
SRPX	1.62088550255325	2.45338338507232	-0.597990514265743	1.07747083873155e-06	5.86731847636545e-05
HSPA7	1.92677187591746	3.01675705082122	-0.646812745350922	1.10548393990721e-06	5.98102472262054e-05
CD79A	4.92027946678903	12.7305788338646	-1.37148585058625	1.15593609553809e-06	6.23387787600154e-05
CXCL11	1.20980406457412	2.51937529913325	-1.05829263654698	1.34890912245508e-06	7.15946322842299e-05
WAS	1.61472238651555	2.47412096364798	-0.61562988885331	1.51284892224516e-06	7.83132781601724e-05
LSP1	3.09736522501363	4.83285156766656	-0.641833177612922	1.69994913570983e-06	8.56202609733492e-05
SLAMF6	0.6234982216057	1.15801852719836	-0.893200985067657	1.7066035380598e-06	8.56980674860446e-05
CCL19	4.6409169786707	13.0182020014135	-1.48804841109671	1.74551506972162e-06	8.71302938969376e-05
CD5	0.989000486862158	1.7549833732869	-0.827414226198515	1.91290847037424e-06	9.46410055018196e-05
APOD	4.88913511566315	8.42582511595392	-0.785238695963168	1.93746523214595e-06	9.52937444972196e-05
BATF2	0.784411033769986	1.19400628260894	-0.60612869359602	1.97090836231469e-06	9.63659028432965e-05
CD2	3.98555121725524	6.85062100155038	-0.781455504423324	2.22713203498685e-06	0.000105221009833238
POU2AF1	0.905108952991925	1.74671762265223	-0.948483025588551	2.29137625217469e-06	0.000107649754906089
GZMH	1.15015275473495	1.9229136690387	-0.741468511102876	2.34503371019301e-06	0.000109556839519101
CD37	1.23873378887029	1.91991313918146	-0.632174864738237	3.58569972013019e-06	0.000156612905484436
BIN2	0.907555452271279	1.38326436325372	-0.608019203628562	3.72426326323673e-06	0.000161822133292763
C1QB	27.4875368934682	41.5621094396135	-0.59649124306552	4.4788419103268e-06	0.000188741549045229
MSC	0.657816976737413	1.07031838757449	-0.702281872520355	4.58934953449653e-06	0.000191951547113655
CSF2RB	1.08763826142954	1.63977958190929	-0.592303092614142	5.24119293735646e-06	0.000209797823258574
NCF1C	0.755074269116352	1.30674660301703	-0.791288948876518	5.55798558798827e-06	0.000220374785536027
NAPSB	2.43588547619777	3.72718962589673	-0.613641913879329	5.98641776557302e-06	0.000232956377643134
RRAD	3.38527692042172	5.79026449048184	-0.774355396722097	6.07108762570057e-06	0.000235704355690394
CXCL10	5.7706130280828	10.6038702217019	-0.877794425033068	7.07269426547357e-06	0.00026656905218095
VPREB3	0.588275063311759	1.14205557934849	-0.957070075164078	7.44784614787748e-06	0.000277589501316002

PI16	1.00387943929258	1.98178341401197	-0.981213281761412	8.88644239140008e-06	0.000322716071205106
CD52	14.392033431629	22.1967873898212	-0.625080442957639	1.19428829153764e-05	0.000416436657498322
CD53	5.63514387202451	8.51412428662169	-0.595405705687576	1.277104906013e-05	0.000438926300894467
PLEK	4.17807170051278	6.28018715129303	-0.587970301541272	1.62676283132205e-05	0.000532891918104169
CCR5	1.10205976823122	1.71558943711887	-0.638501870438692	1.6320277208377e-05	0.00053357444315575
IDO1	0.761682062512327	1.37535993190034	-0.852548395569655	1.8407371283684e-05	0.000588958081040453
CTSW	2.28456529311849	3.46828167667699	-0.602301396357868	2.01378052307503e-05	0.000628959533203248
KLRB1	1.15654730329538	1.82905796703717	-0.661276524077056	2.13802314999049e-05	0.000660385345702404
CYTIP	1.47864965082602	2.29483446043294	-0.634109824739673	2.66607091061832e-05	0.000798488237730186
PTPRC	2.00468419356792	3.12498337074105	-0.640473531959733	2.83125103183848e-05	0.000841414769845367
WIF1	0.360204695460868	2.34762235809112	-2.70431146033176	2.97970846131535e-05	0.000873700529950719
CLEC10A	0.650440456896849	1.05089722948104	-0.692132690708977	3.41019931662172e-05	0.000974375859256892
HLA-DRB6	9.60075385573885	14.7626888278709	-0.620735916596503	3.98985314547926e-05	0.00109419450968806
SAA1	0.701516095007068	1.63407231273612	-1.21992371925487	4.26211305047103e-05	0.00115857633845219
MT1A	1.35904916882704	2.19947414327132	-0.694560989127169	4.86161103598624e-05	0.0012963265547784
SELE	1.24801098590844	2.03778619567832	-0.707372058109629	5.95988514069837e-05	0.00150995760694551
TNFRSF17	0.567958735582707	1.2839768904674	-1.17676121525958	6.55777001904281e-05	0.00163914931087013
PAX9	0.757598829364847	1.23885510871562	-0.709501459118303	7.89149442546377e-05	0.00190883699483943
PODN	0.907672040860992	1.42964207170309	-0.655410972270763	8.45814911433768e-05	0.00202657252779531
TPSAB1	0.832581658435283	1.38173698662322	-0.730819343396853	8.60622166645248e-05	0.00205325106386545
CAPN6	0.704409622727986	1.329555322308	-0.916457285815213	0.000114488636394403	0.00256368946542981
TFF3	33.1619623263594	63.2730790868421	-0.932062424677554	0.000177127000318595	0.00370420704406916
KLHDC7B	0.869162582961458	1.37674710275073	-0.663565598832029	0.000198342492690586	0.00404695898711255
ADRA2A	1.25319207863922	1.91562545747942	-0.612207957688723	0.000212447837691884	0.0042469310295212
HLA-DOA	7.20669549699787	10.8893946862148	-0.595513966678015	0.000355980799779096	0.00634485650785866
C7	4.25034854684148	7.33717214298336	-0.787642980430915	0.000371361587993705	0.00655629110929517
SMR3B	0.681247932660651	2.14740409828042	-1.65634185110491	0.000418093417441062	0.00717000286024693
ANKRD22	0.709385848124797	1.08786965292375	-0.616863250502989	0.000448735989223624	0.00757162978999861
TPSB2	1.59465960701675	2.5607947811158	-0.683343139860437	0.000455731284162669	0.00765884278354337
CD27	9.05501766276338	13.7185465666021	-0.599338280550157	0.000470465367756529	0.00784358364613569
VGF	0.894277608580427	1.90495476194867	-1.09096207900511	0.000551944951738802	0.00894417461890162
CXCL13	3.91915504890135	8.13895902055763	-1.05430163471928	0.000986617628929939	0.0139877870753393
SLC6A14	0.638354980472872	1.14746005351541	-0.846013114796454	0.00107717604803628	0.0150053128552031
LTF	1.95674577202423	2.98811130583212	-0.610777561261136	0.00118885841973676	0.0161012583547126
AREG	1.54115404555294	2.3223703529411	-0.591586986774131	0.00121223475724861	0.0162925305944519
S100B	2.51720108446041	4.04608586653621	-0.684706467709691	0.00160200747290469	0.0203397951064023
NAPSA	2.38000762665874	3.75157662021192	-0.656530826768576	0.00186514726591514	0.0228171042625301
MMP7	6.27668419909388	10.8512708959508	-0.789789491294721	0.0019236573928072	0.0232614144139599
CST1	1.64957194811478	4.11095839819027	-1.31738306808566	0.00196946751855502	0.023644888490483
SFTPB	111.555996942224	180.615773736556	-0.695155822393449	0.00433370665976403	0.0418644356297517
BIRC7	2.58585859403016	4.13984110389744	-0.678932010535374	0.0044482500676253	0.0425747112445546
STMN2	0.891606850565602	1.40164422904739	-0.65264059863507	0.00973519461769488	0.0737816015038312
CR2	0.954286584449515	1.55653878613622	-0.705847030450496	0.0170208538021105	0.10822119842091
C1QL2	1.01526100542538	1.54210200799998	-0.603047534614884	0.03990370459756	0.187154623464842

Up-regulated genes

gene name	DNAm age drift	REST	log2FC	Pvalue	Qvalue
DHRS2	1.03230444031457	0.0549255665861028	4.23224684636633	1.33402851885783e-70	1.37229724633214e-66

AKR1C2	3.69671996538597	0.224878401092519	4.03902875408615	1.63641455560713e-70	1.37229724633214e-66
GPX2	1.00109973388594	0.0240006494475943	5.38236845353921	2.29466496948289e-67	9.62153021704175e-64
AKR1B10	6.93972041826051	0.0711876253980997	6.60710725348711	1.08073755626169e-51	3.62522605872422e-48
AKR1C1	3.72651768419518	0.523494811251087	2.83158096327788	2.06680752792303e-48	5.7774159763875e-45
AKR1C3	5.45676143248165	1.28747504411027	2.08350050305887	3.09494126008042e-41	7.41547925915269e-38
NPY	12.3346352329709	0.40145940086397	4.94131714482576	3.64016681828153e-31	5.08773982301815e-28
THADA	5.7806729646805	2.69425334016315	1.10135194221521	2.63921813205028e-29	3.16178332219624e-26
PDX1	1.16944093540137	0.00184713560143423	9.30631350147363	4.39990324589835e-24	3.883956696853e-21
HHIPL2	1.52127748416874	0.332710128545257	2.19294563631285	4.81043935546889e-17	2.44486935969468e-14
MYH2	1.49699712326867	0.0208184163643471	6.16806731031885	9.05826310124674e-15	3.1651080986273e-12
UCHL1	2.87732071835422	1.511970393613	0.928296148987991	1.08801893415757e-11	2.68356670054277e-09
CYP4F11	2.28919762557597	0.86061783382527	1.41139737418688	1.51173679107973e-11	3.57110555774496e-09
OSGIN1	2.12433228444261	1.24570605511949	0.770045766856189	1.99619359333152e-11	4.6500220760217e-09
KLK4	2.86064868817435	0.986613519793343	1.53578537123616	5.4553962483669e-09	7.03830045212382e-07
SLC38A3	1.47268813258257	0.762109860028538	0.950381060967215	1.95463221188733e-08	2.07487920618824e-06
AKR7A3	1.31092521434989	0.69919314345096	0.90682244263892	3.75323700831636e-08	3.79212597008928e-06
CKMT2	2.98130384583408	1.60574999570935	0.892696125523506	9.8675320482967e-08	8.61970039135585e-06
NPTX2	1.31106418035208	0.740934106813716	0.823321160601065	4.54233672639043e-07	2.99937289665434e-05
PPY	6.49041164406634	0.620940421757264	3.38578322585993	5.06784530193292e-07	3.19540982721875e-05
NQO1	45.0012058397775	27.6986846152936	0.700146194355874	9.29000974445781e-07	5.17647984830719e-05
SLC30A3	1.253708923211	0.590009820882675	1.08739155898129	1.60305504582895e-06	8.19708513068391e-05
HPDL	1.91526232690405	1.15748504008854	0.72654845917062	2.46889859453597e-06	0.00011502324229877
DGAT2	5.44420229617573	3.61578638149577	0.590411228926867	2.78049214774998e-06	0.000127069248779462
C1QL4	3.50093821888384	2.21607510309682	0.659734827373791	3.78296483378054e-06	0.000163105105892461
SLC14A2	1.53015499718879	0.610463225576548	1.32570150353155	4.04850394605135e-06	0.000172777374511891
ECEL1	1.31733250590117	0.69828751319131	0.915726460299963	6.85541907547184e-06	0.000260723557219532
BEX1	20.5391653477424	12.474923691137	0.719346565242387	7.24074429079956e-06	0.000272290948980472
TRIM54	1.44627153418836	0.911490236869917	0.666039331926126	8.22768959961693e-06	0.000302620197291174
CNTFR	3.50429595875934	1.46732930054887	1.25593194487852	1.06710971587864e-05	0.000376790824309823
ACTA1	2.65472820060966	1.10791724197967	1.2607140404356	3.38307847885278e-05	0.000968276318213631
MYL1	1.25743202946284	0.190113952277476	2.7255440982361	9.17084536244563e-05	0.0021568463326072
RPPH1	17.3344696669474	8.5807067011593	1.01447532210187	0.000406197865513757	0.00701622100967737
MSI1	1.15918041359427	0.72116945051317	0.684694934208853	0.000414889241295003	0.00712963356045061
EEF1A2	1.22223546025307	0.714477441258139	0.774561877267327	0.00087269483789717	0.0127055883864682
GATA5	2.30956307857966	1.13241849151686	1.02821273684137	0.00089007616049858	0.0128803773631425
PLA2G2A	3.16846339839765	1.76772426748929	0.841890091480981	0.00177613563247849	0.0219200491743408
FLRT1	2.20858968773394	1.37355541259414	0.685210305948542	0.00249996527920254	0.0284653208844433
GLDN	1.77827469209589	1.10553008301295	0.685739912681504	0.00259456393042313	0.0291405964482914
SH3GL2	1.41007433490911	0.85916932152431	0.714756834356333	0.00290525642880367	0.0318268849274299
PENK	1.78197816955538	0.960026684716777	0.892333250595388	0.00353151523035797	0.0363154956735524
SCARNA10	1.49308024806746	0.825768958676364	0.854481615015921	0.00403123142247871	0.0400069901880549
SST	2.04927724112729	0.879859943097662	1.2197693781934	0.013067524513919	0.0906779152451176
LCN6	6.74198940733992	4.28090959239454	0.655256992745688	0.0138280399475891	0.0942396936208717
KLK1	3.73551429011891	2.08764467253269	0.839430702001721	0.0203396422757953	0.121573941642779
MYOC	1.18749333182813	0.784141279484035	0.598733897634714	0.0257659557617314	0.140984881012521

Table S3. Differentially expressed genes in tumors with highly drifted DNAm age (HDDA).

Down-regulated genes

gene name	DNAm age drift	Others	log2FC	Pvalue	Qvalue
MATN1	0.0462460572176512	1.99970346905352	-5.43431190047848	2.07814277418908e-40	4.3568263260874e-37
IGF2	2.15627997658626	22.1037850896855	-3.35767702195521	4.37484993191997e-30	5.64422946601245e-27
HP	0.123161289616379	1.01505189998598	-3.04293271075457	3.05456006214289e-21	2.13462839009419e-18
MSMP	4.72261462735139	20.1643398264954	-2.09414845392052	2.02821278229437e-17	1.06303702452004e-14
KRT6B	0.11863420296797	1.78667237168815	-3.91268319384599	3.19872270841511e-16	1.49024936848717e-13
KRT6A	0.435503318452817	4.25203850142891	-3.28739904322553	8.77502147672271e-16	3.87301737388403e-13
ALK	0.32566206512111	1.2841848054869	-1.97940525219283	1.34551024586126e-15	5.78638406245772e-13
TCN1	0.146468353790846	1.29587947220915	-3.14527064936585	6.88948413701075e-15	2.51196582491183e-12
BCHE	0.383132964623263	1.3451517764067	-1.81185189921183	2.12179222087791e-12	6.03164392009563e-10
DPRX	0.0135474615396093	4.36102025382782	-8.33049932908969	4.70663665638418e-12	1.21445707693655e-09
IGF1	0.689765506796273	1.5789936340271	-1.1948274640921	1.26840475638276e-11	3.08314269189153e-09
KRT5	0.394864086444318	2.15872062213189	-2.45074848103182	4.09381129412445e-11	8.8027439775712e-09
CD79B	1.28140613193535	2.53918797638956	-0.986639402439997	1.27700455190462e-10	2.47246464377818e-08
S100A2	7.30425135220795	16.2917603123622	-1.15733417741495	1.28252101126104e-10	2.47246464377818e-08
BST2	19.0127725558547	31.6527611470165	-0.735362425205325	3.15127322609e-10	5.87257272755349e-08
NKG7	2.03804139781006	3.92005924943796	-0.943692103398921	3.8078640440564e-10	7.01818634581471e-08
TYMP	3.25425902277418	5.28779211185487	-0.70033637188289	3.95877458033244e-10	7.17008229568538e-08
CCL21	8.05404402248381	30.8144521597027	-1.93582187909644	6.09772366165878e-10	1.03304061872062e-07
PTPRCAP	9.655932891584	18.0503110136237	-0.902536140945524	8.89191123099527e-10	1.43399168429089e-07
CST7	3.77913938551283	7.27676296212065	-0.945239085895908	1.09118099441592e-09	1.72653656965508e-07
KRT14	0.598588741970606	1.88524440866685	-1.65511452033124	2.09245969101468e-09	3.10572866705293e-07
LAG3	0.500883485220174	1.05871842510548	-1.07977199374226	2.11410715530436e-09	3.11033379024252e-07
SFTPA2	2.25074643973019	9.03412346948936	-2.00498109366399	5.94324046993818e-09	7.55151736074267e-07
SLC5A5	1.26357827649044	5.53343183839577	-2.13065947902417	6.69545754480956e-09	8.3803144732497e-07
PDCD1	0.625675497408664	1.28424520350698	-1.03743417371951	7.67773550749142e-09	9.26409927565799e-07
CXCR3	0.826309169736672	1.46205648654269	-0.82324546800771	8.30350400738747e-09	9.80749078957061e-07
PIM2	5.60560277659796	9.81545572294409	-0.80818573635565	8.67125112478467e-09	1.01702254450971e-06
KRT17	3.35439217463965	9.96437710019378	-1.57072825148463	1.03634854008184e-08	1.1905231311132e-06
SLAMF7	1.14841545965586	2.62299848442683	-1.19157231705557	1.37135926452679e-08	1.53336250564289e-06
GZMA	3.94803511487323	7.5129051439079	-0.928236065703254	1.47123449949113e-08	1.63414205466657e-06
CD3D	2.22879272532127	4.51046421538175	-1.01701346861729	1.8044091512529e-08	1.96516560290998e-06
CCL5	10.2178414447259	19.1473535460218	-0.906054549630119	1.82673549230087e-08	1.97664565657227e-06
GZMM	0.641815135409164	1.21667627417925	-0.922715638543377	1.94234831296318e-08	2.07487920618824e-06
ACAP1	0.608800498572895	1.0016422049172	-0.718325812221102	4.21448273986941e-08	4.20379710932532e-06
DPT	3.05450937111354	7.71561181494927	-1.33683989430949	6.85091675055658e-08	6.41919417543771e-06
CHRDL1	0.756602596933855	1.82546126180192	-1.2706534202395	7.30941159905137e-08	6.8107472966272e-06
JSRP1	0.427696800419854	1.07988981927135	-1.33622380266458	8.09774328159619e-08	7.42160384256455e-06
SECTM1	0.877667054200859	1.43775028668866	-0.712067467716439	1.02470433851762e-07	8.8589387451637e-06
FCER1G	10.9139109291289	17.3564364865965	-0.669302598480641	1.12290642777299e-07	9.56009472416676e-06
GZMK	1.58757177983751	3.42346382195307	-1.10863494378299	1.34897319401422e-07	1.10244482043344e-05
CD3E	1.24093702784802	2.29302930935444	-0.885824888639655	1.35454237309775e-07	1.10244482043344e-05
FGL2	2.69095739679722	4.03949671505337	-0.586056006921778	1.56893432917921e-07	1.23540688117341e-05
CHGB	3.96211325248831	10.1367409984921	-1.35525187111983	1.59871192869238e-07	1.24714402176877e-05

C16orf54	0.71358313372947	1.27488160142912	-0.837209848664348	1.5913354799807e-07	1.24714402176877e-05
CCL4	1.46942461059272	2.48193193210664	-0.756212206718542	1.68319914997897e-07	1.30095005269343e-05
CXCL9	12.1852817165268	27.6963041198122	-1.18455386574012	1.82557375471787e-07	1.39175104609673e-05
GBP5	0.693786549732549	1.34500347377134	-0.955046122030545	1.91238697260384e-07	1.45133729884668e-05
CORO1A	3.93109073329537	6.2113230712373	-0.659970946112473	2.08656302061492e-07	1.55898574162614e-05
MYL4	3.73708651206443	6.73061546705898	-0.848824473178478	2.15569463030099e-07	1.59979249289417e-05
CCR7	0.596268258316211	1.09206701799307	-0.873027950678045	2.67048359913157e-07	1.93057547088943e-05
XCL2	0.758109246876989	1.46975996792537	-0.955102895109105	2.79174680184745e-07	2.00099048549511e-05
LCK	0.712590724738028	1.18887028732908	-0.738445707019658	2.86440581343452e-07	2.04433252352867e-05
CYTH4	1.08546731582505	1.67457838670748	-0.625481623858668	3.01034948715251e-07	2.13938905078482e-05
CD48	1.70510098386743	2.91755295181313	-0.774901655439739	3.6331489202195e-07	2.5284304435652e-05
SASH3	3.22821960368514	5.23351785034626	-0.697042295875958	3.91909133350298e-07	2.70497941751078e-05
IL2RB	2.43547171748509	3.98713608927611	-0.711151618744071	4.02881190735813e-07	2.75800952286574e-05
CD8A	1.86986389187489	3.11600694913645	-0.736765191219656	4.4473630049544e-07	2.95996715551965e-05
RUNX3	0.729847112488551	1.21667362504668	-0.7372760276324	4.76710120754447e-07	3.07166872862051e-05
STAT4	0.727146183082054	1.19319080919148	-0.714507437068976	4.75291543109267e-07	3.07166872862051e-05
BATF	1.05394519625869	1.86222488594219	-0.821227455397804	4.86065706101088e-07	3.11156260409444e-05
HSPA6	2.34635237434976	3.75427973485344	-0.67811645765894	6.60617951278784e-07	4.04343680516434e-05
LGALS2	0.715543382061852	1.48688123117898	-1.05517827003976	6.96033519148403e-07	4.19923531768238e-05
PRF1	1.16877784930225	1.79012951158355	-0.615063225264831	7.13227975590034e-07	4.25703188846835e-05
LIMD2	1.2522762113272	1.93936055782144	-0.631028239336705	8.08568908640487e-07	4.69161404099167e-05
HCST	6.42481963363026	9.7656157537953	-0.604055060032376	8.16068636153952e-07	4.70347187820415e-05
IL2RG	2.40472914799067	4.2287479688744	-0.814356171088695	9.22993107638947e-07	5.16014680044014e-05
SRPX	1.62088550255325	2.45338338507232	-0.597990514265743	1.07747083873155e-06	5.86731847636545e-05
HSPA7	1.92677187591746	3.01675705082122	-0.646812745350922	1.10548393990721e-06	5.98102472262054e-05
CD79A	4.92027946678903	12.7305788338646	-1.37148585058625	1.15593609553809e-06	6.23387787600154e-05
CXCL11	1.20980406457412	2.51937529913325	-1.05829263654698	1.34890912245508e-06	7.15946322842299e-05
WAS	1.61472238651555	2.47412096364798	-0.61562988885331	1.51284892224516e-06	7.83132781601724e-05
LSP1	3.09736522501363	4.83285156766656	-0.641833177612922	1.69994913570983e-06	8.56202609733492e-05
SLAMF6	0.6234982216057	1.15801852719836	-0.893200985067657	1.7066035380598e-06	8.56980674860446e-05
CCL19	4.6409169786707	13.0182020014135	-1.48804841109671	1.74551506972162e-06	8.71302938969376e-05
CD5	0.989000486862158	1.7549833732869	-0.827414226198515	1.91290847037424e-06	9.46410055018196e-05
APOD	4.88913511566315	8.42582511595392	-0.785238695963168	1.93746523214595e-06	9.52937444972196e-05
BATF2	0.784411033769986	1.19400628260894	-0.60612869359602	1.97090836231469e-06	9.63659028432965e-05
CD2	3.98555121725524	6.85062100155038	-0.781455504423324	2.22713203498685e-06	0.000105221009833238
POU2AF1	0.905108952991925	1.74671762265223	-0.948483025588551	2.29137625217469e-06	0.000107649754906089
GZMH	1.15015275473495	1.9229136690387	-0.741468511102876	2.34503371019301e-06	0.000109556839519101
CD37	1.23873378887029	1.91991313918146	-0.632174864738237	3.58569972013019e-06	0.000156612905484436
BIN2	0.907555452271279	1.38326436325372	-0.608019203628562	3.72426326323673e-06	0.000161822133292763
C1QB	27.4875368934682	41.5621094396135	-0.59649124306552	4.4788419103268e-06	0.000188741549045229
MSC	0.657816976737413	1.07031838757449	-0.702281872520355	4.58934953449653e-06	0.000191951547113655
CSF2RB	1.08763826142954	1.63977958190929	-0.592303092614142	5.24119293735646e-06	0.000209797823258574
NCF1C	0.755074269116352	1.30674660301703	-0.791288948876518	5.55798558798827e-06	0.000220374785536027
NAPSB	2.43588547619777	3.72718962589673	-0.613641913879329	5.98641776557302e-06	0.000232956377643134
RRAD	3.38527692042172	5.79026449048184	-0.774355396722097	6.07108762570057e-06	0.000235704355690394
CXCL10	5.7706130280828	10.6038702217019	-0.877794425033068	7.07269426547357e-06	0.00026656905218095
VPREB3	0.588275063311759	1.14205557934849	-0.957070075164078	7.44784614787748e-06	0.000277589501316002

PI16	1.00387943929258	1.98178341401197	-0.981213281761412	8.88644239140008e-06	0.000322716071205106
CD52	14.392033431629	22.1967873898212	-0.625080442957639	1.19428829153764e-05	0.000416436657498322
CD53	5.63514387202451	8.51412428662169	-0.595405705687576	1.277104906013e-05	0.000438926300894467
PLEK	4.17807170051278	6.28018715129303	-0.587970301541272	1.62676283132205e-05	0.000532891918104169
CCR5	1.10205976823122	1.71558943711887	-0.638501870438692	1.6320277208377e-05	0.00053357444315575
IDO1	0.761682062512327	1.37535993190034	-0.852548395569655	1.8407371283684e-05	0.000588958081040453
CTSW	2.28456529311849	3.46828167667699	-0.602301396357868	2.01378052307503e-05	0.000628959533203248
KLRB1	1.15654730329538	1.82905796703717	-0.661276524077056	2.13802314999049e-05	0.000660385345702404
CYTIP	1.47864965082602	2.29483446043294	-0.634109824739673	2.66607091061832e-05	0.000798488237730186
PTPRC	2.00468419356792	3.12498337074105	-0.640473531959733	2.83125103183848e-05	0.000841414769845367
WIF1	0.360204695460868	2.34762235809112	-2.70431146033176	2.97970846131535e-05	0.000873700529950719
CLEC10A	0.650440456896849	1.05089722948104	-0.692132690708977	3.41019931662172e-05	0.000974375859256892
HLA-DRB6	9.60075385573885	14.7626888278709	-0.620735916596503	3.98985314547926e-05	0.00109419450968806
SAA1	0.701516095007068	1.63407231273612	-1.21992371925487	4.26211305047103e-05	0.00115857633845219
MT1A	1.35904916882704	2.19947414327132	-0.694560989127169	4.86161103598624e-05	0.0012963265547784
SELE	1.24801098590844	2.03778619567832	-0.707372058109629	5.95988514069837e-05	0.00150995760694551
TNFRSF17	0.567958735582707	1.2839768904674	-1.17676121525958	6.55777001904281e-05	0.00163914931087013
PAX9	0.757598829364847	1.23885510871562	-0.709501459118303	7.89149442546377e-05	0.00190883699483943
PODN	0.907672040860992	1.42964207170309	-0.655410972270763	8.45814911433768e-05	0.00202657252779531
TPSAB1	0.832581658435283	1.38173698662322	-0.730819343396853	8.60622166645248e-05	0.00205325106386545
CAPN6	0.704409622727986	1.329555322308	-0.916457285815213	0.000114488636394403	0.00256368946542981
TFF3	33.1619623263594	63.2730790868421	-0.932062424677554	0.000177127000318595	0.00370420704406916
KLHDC7B	0.869162582961458	1.37674710275073	-0.663565598832029	0.000198342492690586	0.00404695898711255
ADRA2A	1.25319207863922	1.91562545747942	-0.612207957688723	0.000212447837691884	0.0042469310295212
HLA-DOA	7.20669549699787	10.8893946862148	-0.595513966678015	0.000355980799779096	0.00634485650785866
C7	4.25034854684148	7.33717214298336	-0.787642980430915	0.000371361587993705	0.00655629110929517
SMR3B	0.681247932660651	2.14740409828042	-1.65634185110491	0.000418093417441062	0.00717000286024693
ANKRD22	0.709385848124797	1.08786965292375	-0.616863250502989	0.000448735989223624	0.00757162978999861
TPSB2	1.59465960701675	2.5607947811158	-0.683343139860437	0.000455731284162669	0.00765884278354337
CD27	9.05501766276338	13.7185465666021	-0.599338280550157	0.000470465367756529	0.00784358364613569
VGF	0.894277608580427	1.90495476194867	-1.09096207900511	0.000551944951738802	0.00894417461890162
CXCL13	3.91915504890135	8.13895902055763	-1.05430163471928	0.000986617628929939	0.0139877870753393
SLC6A14	0.638354980472872	1.14746005351541	-0.846013114796454	0.00107717604803628	0.0150053128552031
LTF	1.95674577202423	2.98811130583212	-0.610777561261136	0.00118885841973676	0.0161012583547126
AREG	1.54115404555294	2.3223703529411	-0.591586986774131	0.00121223475724861	0.0162925305944519
S100B	2.51720108446041	4.04608586653621	-0.684706467709691	0.00160200747290469	0.0203397951064023
NAPSA	2.38000762665874	3.75157662021192	-0.656530826768576	0.00186514726591514	0.0228171042625301
MMP7	6.27668419909388	10.8512708959508	-0.789789491294721	0.0019236573928072	0.0232614144139599
CST1	1.64957194811478	4.11095839819027	-1.31738306808566	0.00196946751855502	0.023644888490483
SFTPB	111.555996942224	180.615773736556	-0.695155822393449	0.00433370665976403	0.0418644356297517
BIRC7	2.58585859403016	4.13984110389744	-0.678932010535374	0.0044482500676253	0.0425747112445546
STMN2	0.891606850565602	1.40164422904739	-0.65264059863507	0.00973519461769488	0.0737816015038312
CR2	0.954286584449515	1.55653878613622	-0.705847030450496	0.0170208538021105	0.10822119842091
C1QL2	1.01526100542538	1.54210200799998	-0.603047534614884	0.03990370459756	0.187154623464842

Up-regulated genes

gene name	DNAm age drift	REST	log2FC	Pvalue	Qvalue
DHRS2	1.03230444031457	0.0549255665861028	4.23224684636633	1.33402851885783e-70	1.37229724633214e-66

AKR1C2	3.69671996538597	0.224878401092519	4.03902875408615	1.63641455560713e-70	1.37229724633214e-66
GPX2	1.00109973388594	0.0240006494475943	5.38236845353921	2.29466496948289e-67	9.62153021704175e-64
AKR1B10	6.93972041826051	0.0711876253980997	6.60710725348711	1.08073755626169e-51	3.62522605872422e-48
AKR1C1	3.72651768419518	0.523494811251087	2.83158096327788	2.06680752792303e-48	5.7774159763875e-45
AKR1C3	5.45676143248165	1.28747504411027	2.08350050305887	3.09494126008042e-41	7.41547925915269e-38
NPY	12.3346352329709	0.40145940086397	4.94131714482576	3.64016681828153e-31	5.08773982301815e-28
THADA	5.7806729646805	2.69425334016315	1.10135194221521	2.63921813205028e-29	3.16178332219624e-26
PDX1	1.16944093540137	0.00184713560143423	9.30631350147363	4.39990324589835e-24	3.883956696853e-21
HHIPL2	1.52127748416874	0.332710128545257	2.19294563631285	4.81043935546889e-17	2.44486935969468e-14
MYH2	1.49699712326867	0.0208184163643471	6.16806731031885	9.05826310124674e-15	3.1651080986273e-12
UCHL1	2.87732071835422	1.511970393613	0.928296148987991	1.08801893415757e-11	2.68356670054277e-09
CYP4F11	2.28919762557597	0.86061783382527	1.41139737418688	1.51173679107973e-11	3.57110555774496e-09
OSGIN1	2.12433228444261	1.24570605511949	0.770045766856189	1.99619359333152e-11	4.6500220760217e-09
KLK4	2.86064868817435	0.986613519793343	1.53578537123616	5.4553962483669e-09	7.03830045212382e-07
SLC38A3	1.47268813258257	0.762109860028538	0.950381060967215	1.95463221188733e-08	2.07487920618824e-06
AKR7A3	1.31092521434989	0.69919314345096	0.90682244263892	3.75323700831636e-08	3.79212597008928e-06
CKMT2	2.98130384583408	1.60574999570935	0.892696125523506	9.8675320482967e-08	8.61970039135585e-06
NPTX2	1.31106418035208	0.740934106813716	0.823321160601065	4.54233672639043e-07	2.99937289665434e-05
PPY	6.49041164406634	0.620940421757264	3.38578322585993	5.06784530193292e-07	3.19540982721875e-05
NQO1	45.0012058397775	27.6986846152936	0.700146194355874	9.29000974445781e-07	5.17647984830719e-05
SLC30A3	1.253708923211	0.590009820882675	1.08739155898129	1.60305504582895e-06	8.19708513068391e-05
HPDL	1.91526232690405	1.15748504008854	0.72654845917062	2.46889859453597e-06	0.00011502324229877
DGAT2	5.44420229617573	3.61578638149577	0.590411228926867	2.78049214774998e-06	0.000127069248779462
C1QL4	3.50093821888384	2.21607510309682	0.659734827373791	3.78296483378054e-06	0.000163105105892461
SLC14A2	1.53015499718879	0.610463225576548	1.32570150353155	4.04850394605135e-06	0.000172777374511891
ECEL1	1.31733250590117	0.69828751319131	0.915726460299963	6.85541907547184e-06	0.000260723557219532
BEX1	20.5391653477424	12.474923691137	0.719346565242387	7.24074429079956e-06	0.000272290948980472
TRIM54	1.44627153418836	0.911490236869917	0.666039331926126	8.22768959961693e-06	0.000302620197291174
CNTFR	3.50429595875934	1.46732930054887	1.25593194487852	1.06710971587864e-05	0.000376790824309823
ACTA1	2.65472820060966	1.10791724197967	1.2607140404356	3.38307847885278e-05	0.000968276318213631
MYL1	1.25743202946284	0.190113952277476	2.7255440982361	9.17084536244563e-05	0.0021568463326072
RPPH1	17.3344696669474	8.5807067011593	1.01447532210187	0.000406197865513757	0.00701622100967737
MSI1	1.15918041359427	0.72116945051317	0.684694934208853	0.000414889241295003	0.00712963356045061
EEF1A2	1.22223546025307	0.714477441258139	0.774561877267327	0.00087269483789717	0.0127055883864682
GATA5	2.30956307857966	1.13241849151686	1.02821273684137	0.00089007616049858	0.0128803773631425
PLA2G2A	3.16846339839765	1.76772426748929	0.841890091480981	0.00177613563247849	0.0219200491743408
FLRT1	2.20858968773394	1.37355541259414	0.685210305948542	0.00249996527920254	0.0284653208844433
GLDN	1.77827469209589	1.10553008301295	0.685739912681504	0.00259456393042313	0.0291405964482914
SH3GL2	1.41007433490911	0.85916932152431	0.714756834356333	0.00290525642880367	0.0318268849274299
PENK	1.78197816955538	0.960026684716777	0.892333250595388	0.00353151523035797	0.0363154956735524
SCARNA10	1.49308024806746	0.825768958676364	0.854481615015921	0.00403123142247871	0.0400069901880549
SST	2.04927724112729	0.879859943097662	1.2197693781934	0.013067524513919	0.0906779152451176
LCN6	6.74198940733992	4.28090959239454	0.655256992745688	0.0138280399475891	0.0942396936208717
KLK1	3.73551429011891	2.08764467253269	0.839430702001721	0.0203396422757953	0.121573941642779
MYOC	1.18749333182813	0.784141279484035	0.598733897634714	0.0257659557617314	0.140984881012521
